# eFORGE: A Tool for Identifying Cell Type-Specific Signal in Epigenomic Data

**DOI:** 10.1016/j.celrep.2016.10.059

**Published:** 2016-11-15

**Authors:** Charles E. Breeze, Dirk S. Paul, Jenny van Dongen, Lee M. Butcher, John C. Ambrose, James E. Barrett, Robert Lowe, Vardhman K. Rakyan, Valentina Iotchkova, Mattia Frontini, Kate Downes, Willem H. Ouwehand, Jonathan Laperle, Pierre-Étienne Jacques, Guillaume Bourque, Anke K. Bergmann, Reiner Siebert, Edo Vellenga, Sadia Saeed, Filomena Matarese, Joost H.A. Martens, Hendrik G. Stunnenberg, Andrew E. Teschendorff, Javier Herrero, Ewan Birney, Ian Dunham, Stephan Beck

**Affiliations:** 1 UCL Cancer Institute, University College London, London WC1E 6BT, UK; 2 Department of Biological Psychology, Vrije Universiteit Amsterdam, 1081BT Amsterdam, the Netherlands; 3 Department of Surgery and Cancer, Imperial College London, London W12 0NN, UK; 4 Blizard Institute, Barts and The London School of Medicine and Dentistry, Queen Mary University of London, E1 2AT London, UK; 5 European Molecular Biology Laboratory, European Bioinformatics Institute (EMBL-EBI), Wellcome Genome Campus, Hinxton, Cambridge CB10 1SD, UK; 6 Department of Human Genetics, The Wellcome Trust Sanger Institute, Wellcome Genome Campus, Hinxton, Cambridge CB10 1HH, UK; 7 Department of Haematology, University of Cambridge, Cambridge Biomedical Campus, Long Road, Cambridge CB2 0PT, UK; 8 National Health Service (NHS) Blood and Transplant, University of Cambridge, Cambridge Biomedical Campus, Long Road, Cambridge CB2 0PT, UK; 9 British Heart Foundation Centre of Excellence, Cambridge Biomedical Campus, Long Road, Cambridge CB2 0QQ, UK; 10 Département d’Informatique, Université de Sherbrooke, Sherbrooke, QC J1K 2R1, Canada; 11 Département de Biologie, Université de Sherbrooke, Sherbrooke, QC J1K 2R1, Canada; 12 Centre de recherche du Centre hospitalier universitaire de Sherbrooke, Sherbrooke, QC J1H 5N4, Canada; 13 Department of Human Genetics, McGill University, Montréal, QC H3G 1Y6, Canada; 14 Génome Québec Innovation Center, Montréal, QC H3A 0G1, Canada; 15 Institute of Human Genetics, Christian Albrechts University, 24105 Kiel, Germany; 16 Department of Pediatrics, Christian-Albrechts-University Kiel & University Hospital Schleswig-Holstein, 24105 Kiel, Germany; 17 Institute of Human Genetics, University of Ulm, Albert-Einstein-Allee 11, 89081 Ulm, Germany; 18 Department of Hematology, University of Groningen and University Medical Center Groningen, PO Box 30001, 9700 RB Groningen, the Netherlands; 19 Department of Biochemistry, PMAS Arid Agriculture University Rawalpindi, 46300 Rawalpindi, Pakistan; 20 Department of Molecular Biology, Faculty of Science, Nijmegen Centre for Molecular Life Sciences, Radboud University, 6500 HB Nijmegen, the Netherlands

**Keywords:** bioinformatics, epigenetics, epigenome-wide association study, histone marks, DNase I hypersensitive sites

## Abstract

Epigenome-wide association studies (EWAS) provide an alternative approach for studying human disease through consideration of non-genetic variants such as altered DNA methylation. To advance the complex interpretation of EWAS, we developed eFORGE (http://eforge.cs.ucl.ac.uk/), a new standalone and web-based tool for the analysis and interpretation of EWAS data. eFORGE determines the cell type-specific regulatory component of a set of EWAS-identified differentially methylated positions. This is achieved by detecting enrichment of overlap with DNase I hypersensitive sites across 454 samples (tissues, primary cell types, and cell lines) from the ENCODE, Roadmap Epigenomics, and BLUEPRINT projects. Application of eFORGE to 20 publicly available EWAS datasets identified disease-relevant cell types for several common diseases, a stem cell-like signature in cancer, and demonstrated the ability to detect cell-composition effects for EWAS performed on heterogeneous tissues. Our approach bridges the gap between large-scale epigenomics data and EWAS-derived target selection to yield insight into disease etiology.

## Highlights

• Development of a tool for the analysis and interpretation of EWAS data
• Identification of cell type-specific signals in heterogeneous EWAS data
• Identification of cell-composition effects in EWAS
• Compilation of eFORGE catalog of 20 published EWAS

## Graphical Abstract

**Figure:**
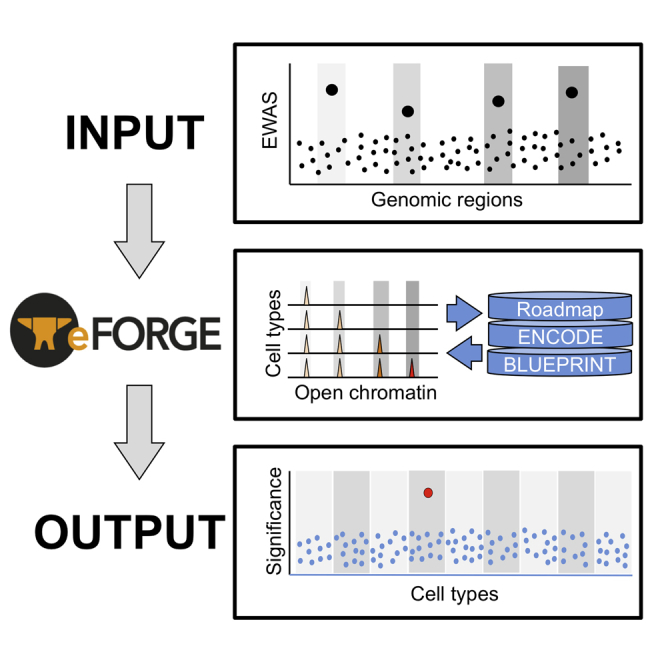

## Teaser

Breeze et al. develop a tool for the analysis and interpretation of EWAS data. The eFORGE tool identifies cell type-specific, disease-relevant signals in heterogeneous EWAS data and can also identify cell-composition effects. Explore consortium data at the Cell Press IHEC webportal at http://www.cell.com/consortium/IHEC.

## Introduction

Common complex diseases, such as autoimmune, metabolic, and neurodegenerative diseases as well as cancer, typically involve multiple genetic and non-genetic factors. The search for genetic factors through genome-wide association studies (GWAS) has identified thousands of replicated SNPs associated with many diseases and other complex phenotypes (Welter et al., 2014). Notably, most of the identified variants were found to map at non-protein coding regions where their molecular consequences are difficult to evaluate (Paul et al., 2014). The search for non-genetic factors is more complicated due to the many confounding factors, in particular, genetic heterogeneity between individuals and cell-type heterogeneity in accessible sample material (e.g., whole blood). Nonetheless, significant progress has been made following the introduction of systematic epigenome-wide association studies (EWAS) in 2011 (Rakyan et al., 2011), including correction of cell-composition effects (Houseman et al., 2012, Zou et al., 2014, McGregor et al., 2016, Houseman et al., 2016). Since then, over 250 EWAS have been conducted (Michels et al., 2013). A large proportion of these EWAS have correlated DNA methylation (DNAm) profiles with disease status and environmental exposure in cross-sectional case-control cohorts. A subset of these studies reported differentially methylated positions (DMPs) that replicated in independent sample cohorts, e.g., for smoking behavior (Philibert et al., 2015). Fine mapping of the disease-associated genetic variants and integrative analysis with cell type-specific epigenomic datasets suggest a possible causal involvement of affected regulatory sequences, especially enhancers (Farh et al., 2015, Kundaje et al., 2015).

Several large-scale initiatives such as the ENCODE (ENCODE Project Consortium, 2012), Epigenomics Roadmap (Kundaje et al., 2015), and BLUEPRINT (Adams et al., 2012) projects have mapped gene regulatory elements across a wide range of tissues and cell types using a variety of assays. Active regulatory elements have been shown to concentrate at DNase I hypersensitive sites (DHSs) (Dorschner et al., 2004). The intersection of these DHSs with common disease-associated variants has proved to be a powerful approach to identify potential regulatory variants implicated in the disease (Maurano et al., 2012). This approach has been implemented as a publicly available web tool, termed FORGE, and systematically applied to analyze SNP sets for 260 phenotypes from the GWAS catalog (Dunham et al., 2015). The functional interpretation of the resulting SNP-DHS overlaps is that highly associated GWAS SNPs are enriched for DHSs of specific cell types that are relevant to the etiology of the disease under investigation. This is because such SNPs are thought to exert functional changes in affected regulatory elements (marked by DHSs), which, in turn, can then affect gene expression and may ultimately result in phenotypic changes.

While genotypes are generally invariable, epigenotypes are ontogenetically, spatially, and temporally variable. This can impede the meaningful interpretation of DMPs identified through EWAS (Michels et al., 2013, Birney et al., 2016, Paul and Beck, 2014). For example, EWAS are often performed on heterogeneous tissues (e.g., whole blood) where cell-composition effects, or only a specific cell type within that tissue, may be driving the observed epigenetic signal. While significant progress has been made to reduce major sources of confounding (reviewed in Liang and Cookson, 2014, Wilhelm-Benartzi et al., 2013, Rakyan et al., 2011, Michels et al., 2013, Birney et al., 2016), additional tools are required to improve EWAS analysis and elucidate disease mechanisms. To this end, we have developed eFORGE (experimentally derived Functional element Overlap analysis of ReGions from EWAS), a bioinformatics tool that informs which EWAS DMPs are likely to be functional and in which tissue or cell type. This is achieved by overlap analysis between a DMP set of choice and reference DHS maps, improving on previous overlap analyses (Stadler et al., 2011, Slieker et al., 2013). eFORGE has been implemented both as standalone software and an interactive web tool, and rigorously tested for performance criteria, including reproducibility, false-positive rates, and code execution speed. To demonstrate its utility, we systematically analyzed publicly available EWAS datasets to explore the suitability of eFORGE for analyzing surrogate tissues and detecting cell-composition effects, with the ultimate aim of providing insights into disease mechanisms.

## Results

### eFORGE Method and Workflow

**Figure 1:**
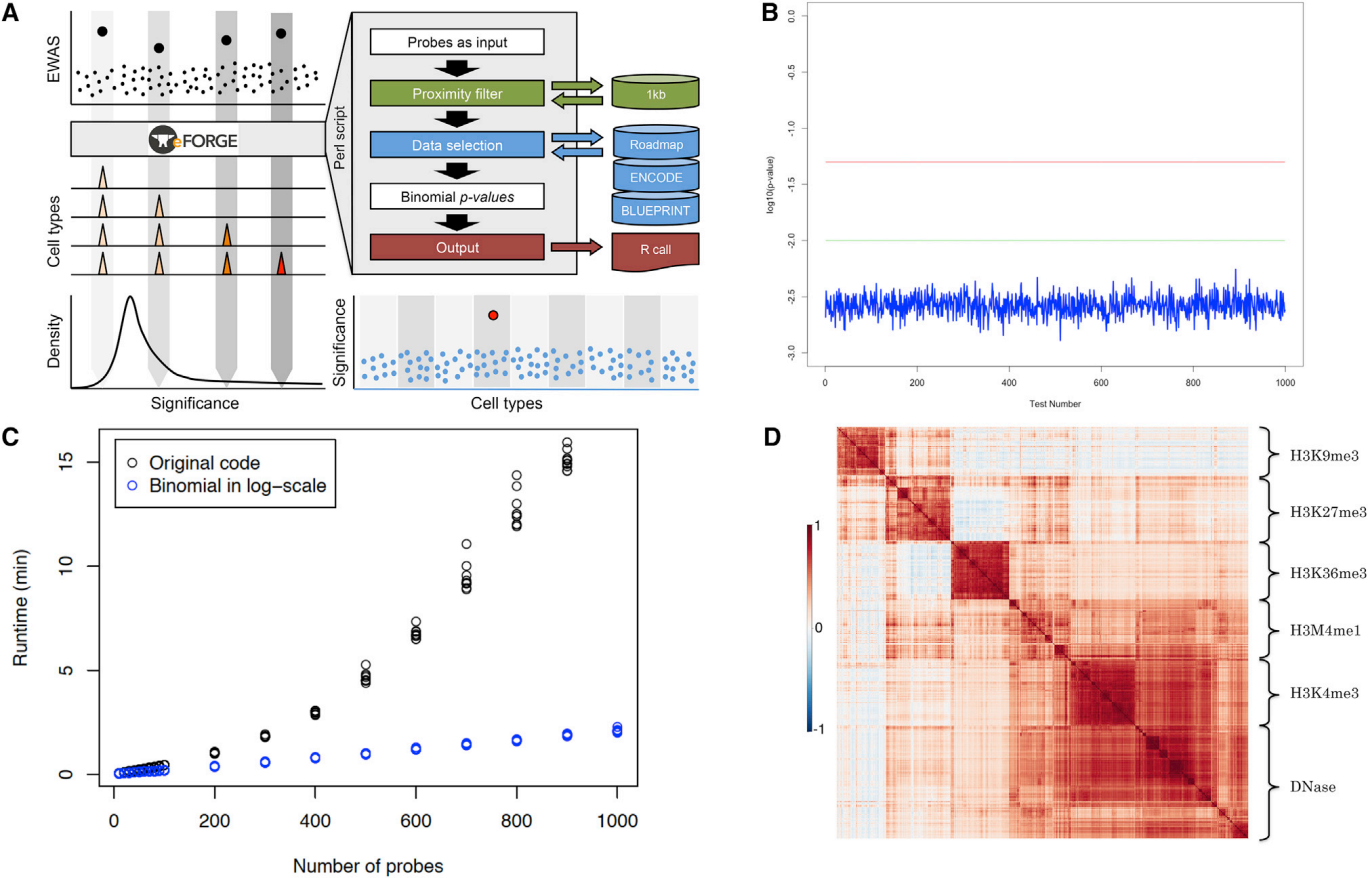
eFORGE Overview and Performance (A) Concept, components, and flowchart of eFORGE: upper-left panel depicts typical EWAS results with top hits marked as large black dots that serve as input for eFORGE. The main components of eFORGE are controlled by Perl software that uses data from the Roadmap Epigenomics, ENCODE, and BLUEPRINT projects to compute enrichment and significance profiles (illustrated by middle and bottom left panels). R code is used to generate output graphs (illustrated by bottom right panel) with predicted target cell types marked in red. (B) Reproducibility: using the CD14^+^ tDMP dataset (Jaffe and Irizarry, 2014), 1,000 different runs were performed showing that the variability due to random background sampling is well below the two eFORGE thresholds (green and red lines) that affect target prediction (shown in log scale). These data indicate high reproducibility between eFORGE runs. (C) Runtime: comparison of Perl BigFloat and BigInt (original code, in black) versus logarithm-based code (in blue) for the management of decimal p value numbers shows up to a 15-fold increase in speed for logarithm-based code. Original code was unable to process 1,000 probes, so data are only shown for probe sets under 1,000 probes. (D) GeEC correlation data matrix for DHS/Histone mark data from the Roadmap, ENCODE, and BLUEPRINT projects. Red regions show high positive correlation (as measured by Pearson correlation coefficient), white regions show no correlation and blue regions show high negative correlation. Grouping of data by hierarchical clustering agrees with original DHS/Histone mark label (y axis), suggesting a similarity in measurements between different consortia. See also Tables S1–S5, S6 and Data S1.

The main objective of eFORGE is the prediction of disease-relevant cell type(s) from EWAS data generated in heterogeneous tissues, as illustrated in Figure 1A. eFORGE analyses such EWAS data generated using Illumina Infinium BeadChips (i.e., 27k, 450k, and EPIC arrays). For a given set of high-scoring probes on the array platform (indicated by large black dots in upper left panel), eFORGE generates a background of 1,000 random probe sets with matching properties regarding their location within genes and CpG islands (Tables S1 and S2). It then overlaps these sets with DHSs for up to 454 selectable reference samples (tissues, primary cell types, and cell lines) from the ENCODE, Epigenomics Roadmap, and BLUEPRINT projects (Data S1). Finally, eFORGE compares the number of DMPs overlapping DHSs with those obtained by random probes to calculate enrichment scores for each of the selected cell types. For a given eFORGE plot, as illustrated in the bottom right panel of Figure 1A, cell types are shown on the horizontal axis and the significance (e.g., –log10 binomial p value) is shown on the vertical axis. Each dot represents an enrichment p value for a given cell type. Enrichments with multiple testing-corrected q values <0.05 and <0.01 are shown as pink and red dots, respectively. Samples that do not reach statistical significance (q value >0.05) are shown in blue.

### Assessment of eFORGE Performance

First, we assessed the reproducibility of eFORGE. Each time eFORGE is run on a dataset, it selects matched background probes at random. Because of this, the background is different for each eFORGE analysis. To assess the level of reproducibility between individual analyses, we compared 1,000 independent eFORGE runs using the same dataset, which also represents the default test set made available as part of the stand-alone and web-based versions of eFORGE (see also Experimental Procedures). Using this test set, we obtained a highly consistent pattern of enrichment for the same target cell types between individual runs (Figure 1B), demonstrating that eFORGE results are highly reproducible.

Second, we assessed two methods for the correction of multiple testing: Benjamini-Hochberg (BH), a false discovery rate (FDR)-based correction method for independent tests (Benjamini and Hochberg, 1995), and Benjamini-Yekutieli (BY), an FDR-based correction for multiple tests that are not independent (Benjamini and Yekutieli, 2001). We reasoned that the latter would be more appropriate in our case as DHSs across different cell types have been found to be cell lineage dependent (Stergachis et al., 2013). Benjamini-Hochberg correction resulted in one false-positive (q value <0.01), after running >2.3 million tests (8,000 runs of 299 samples each), compared to no false-positives with BY correction (q value <0.01). Based on this result, we implemented BY correction for eFORGE analyses (see also Tables S3 and S4).

Third, we assessed eFORGE computational speed using different approaches to manage the large decimal p value numbers resulting from the many statistical tests performed. The Perl BigFloat and BigInt functions used by FORGE (Dunham et al., 2015) were slow and, in some cases, even impeded eFORGE code execution. Switching to using the logarithms of the values for the binomial test not only reduced the amount of digits needed in calculation, but also dramatically increased code execution speed as shown in Figure 1C. We anticipate that this advance will become particularly noticeable in the online version of eFORGE when encountering high user demand.

#### Inter-consortium Correlation Analysis

One possible caveat when interpreting eFORGE results could be consortium-specific differences in the generation of reference datasets used by eFORGE. These include data-analysis pipelines, experimental protocols (including read depth specifications), and materials used (i.e., ENCODE predominantly analyzed transformed cell lines, whereas Roadmap and BLUEPRINT used primary tissue samples). In order to quantify any consortium bias on data generation, we applied the Genomic Efficient Correlator (GeEC) tool (see Experimental Procedures). This tool can measure the correlation of data from different consortia and identify drivers of clustering for these data. This analysis did not detect any consortium bias on data generation (Figure 1D; Table S5).

#### Application to tDMPs

**Figure 2:**
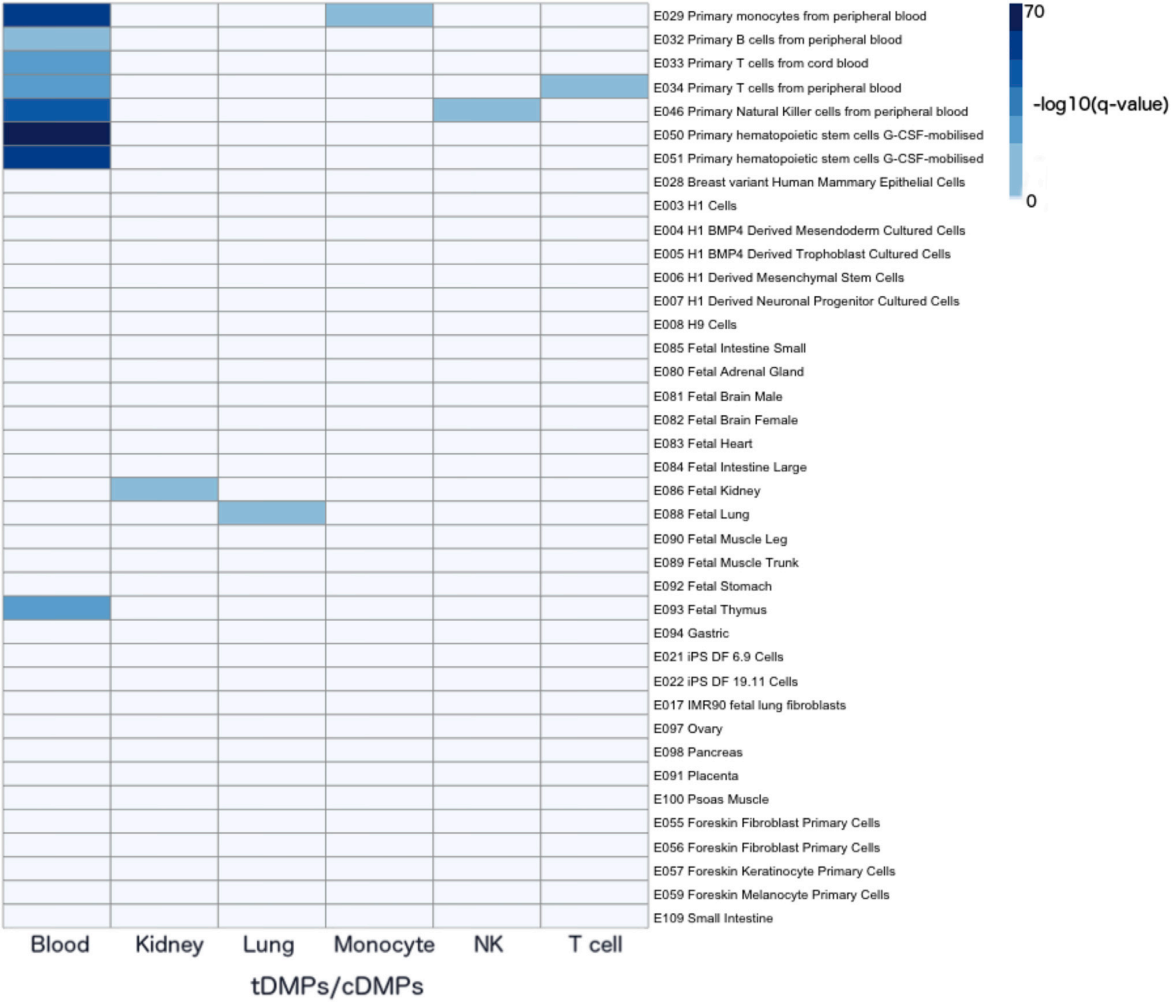
eFORGE Analysis of tDMPs and cDMPs Results show ability to predict target tissues from known tissue-specific differentially methylated positions (tDMPs) and cell type-specific DMPs (cDMPs): the heatmap is a composite of results for the top 1,000 tDMPs for blood, kidney, and lung (Lowe et al., 2015), and top cDMPs for CD14^+^, T cells, and NK cells (Jaffe and Irizarry, 2014). With tDMPs and cDMPs, we have the advantage of a known prior tissue- or cell type-specific association. We can thus test whether the eFORGE tool identifies the correct tissue. The color-coded enrichment results show that eFORGE identified the correct tissue or cell type each time, with no false-positives. This confirms the tool can signal when regions are associated by DNAm with a specific cell type. See also Figure S1 and Data S2, S3, S4, and S5.

As a positive control, we assessed the ability of eFORGE to identify the correct tissues and cell type(s) when tested with probe sets of established tissue and cell-type specificity. In this regard, we analyzed three sets of previously reported tissue-specific DMPs (tDMPs) (Lowe et al., 2015) and three sets of cell type-specific DMPs (cDMPs) (Jaffe and Irizarry, 2014) using consolidated Roadmap DHS data. Figure 2 shows the resulting heatmap, which demonstrates the ability of eFORGE to unambiguously identify the relevant tissues and cell types for each tDMP and cDMP set (i.e., blood, kidney, lung, monocytes, natural killer cells, and T cells). To quantify the level at which eFORGE can detect mixed tissue- and cell type-specific enrichment, we next assessed its performance on mixed tDMP and cDMP probe lists. Figure S1A shows the result for a mixture of tDMPs from lung and kidney tissues (Lowe et al., 2015). Although both tissues were predicted correctly in eFORGE, the tissue-specific signal was lower, due to a lack of specific enrichment for the mixed sets in each of the cell types. Figure S1B shows the corresponding results for mixed cDMPs. Here, sets of 148 B cell-specific and 148 monocyte-specific cDMPs (Jaffe and Irizarry, 2014) were mixed, and again the corresponding cell types were correctly predicted. Taken together, we provided evidence that eFORGE can identify the correct target tissues and cell types from individual and mixed probe sets.

### Application to EWAS Data

**Figure 3:**
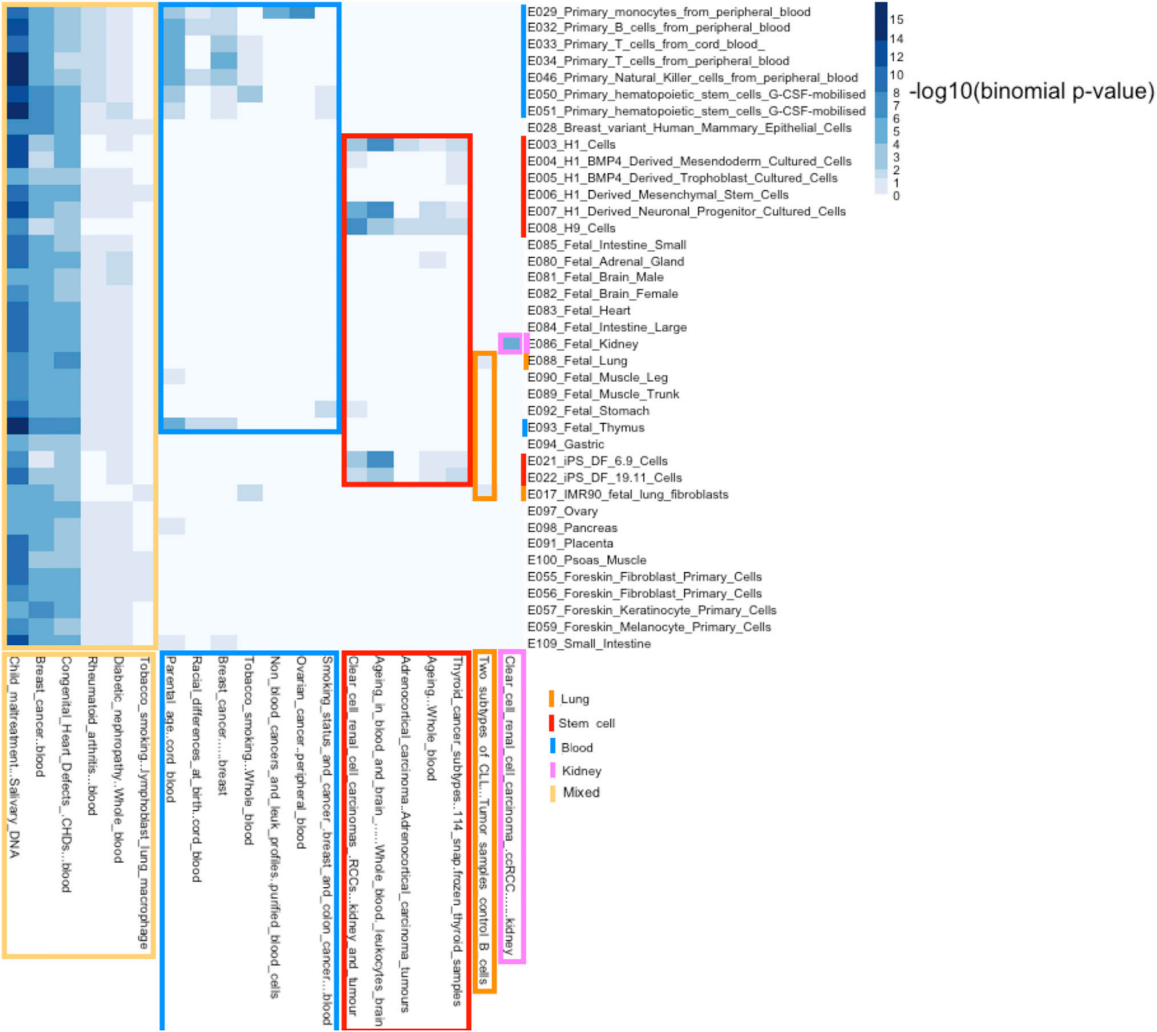
Aggregated Enrichment Statistics for Studies with eFORGE Signal from a Recent Review Studies were obtained from the review by Michels et al. (2013). This heatmap shows the enrichment statistics (presented as –log10(binomial p value)) for an unbiased selection of EWAS (n = 20 studies, each with at least 100 samples). Many of these studies show an enrichment pattern specific to particular tissues, such as blood (blue box, seven studies) and stem cells (red box, five studies). In addition, one ccRCC study shows a kidney specific enrichment and one CLL study presents a lung-specific enrichment (lung tissue and IMR90). Other patterns are more mixed (yellow box, six studies). Of the seven blood-enriched studies, six were performed in blood and one was performed in breast cancer tissue, which may contain immune cells. All five studies that show a stem cell-specific enrichment are exclusively cancer or aging EWAS. Of the six studies that show a mixed enrichment, there is evidence of different components underlying variation. For example, the EWAS on child maltreatment performed on salivary DNA, despite showing enrichment for many tissues, has blood cell types as the highest categories. Work remains to be done to refine these mixed signals and define the components that are driving enrichment for several different tissue types. See also Table S7.

**Figure 4:**
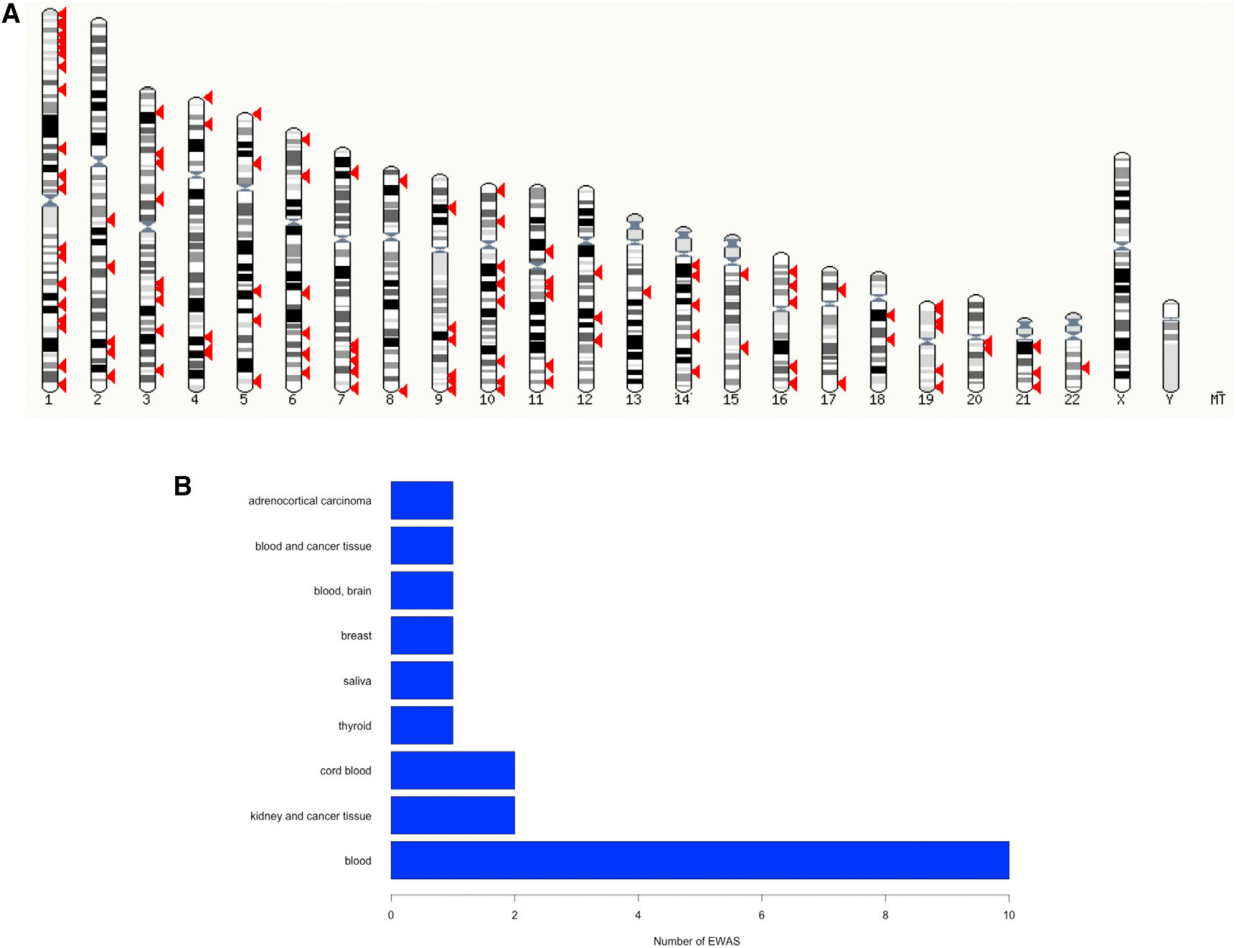
Karyotype View of EWAS Hits and Bar Chart of EWAS Tissues (A) This karyotype view was obtained taking top ten study hits from each of the 20 EWAS with eFORGE signal (taken from Michels et al., 2013) and performed using ensembl KaryoView (http://www.ensembl.org/Homo_sapiens/Location/Genome). Many EWAS exclude probes from sex chromosomes as part of study analysis, and therefore there is an absence of top hits in these chromosomes on the graph. Apart from this, there seems to be no strong bias in the distribution of EWAS hits along the genome. (B) Bar chart indicating analyzed tissue for 20 EWAS with eFORGE signal from Michels et al. (2013). As is to be expected for an easily accessible tissue, blood is the most analyzed category, with ten studies. See also Table S7.

Next, we applied eFORGE to analyze published EWAS data. First, we considered all EWAS compiled in a review article (Michels et al., 2013) that analyzed at least 100 samples using Illumina Infinium BeadChips. This qualified 44 datasets for eFORGE analysis, of which 20 showed eFORGE signal (q value <0.05). 14 studies showed an enrichment pattern specific to particular tissues. For instance, we observed blood-specific patterns for six blood-based EWAS, and stem cell-specific patterns for five cancer and aging EWAS (Figure 3). The genome-wide distribution of hits from these studies is shown in Figure 4. In addition, we found a larger average sample size for studies that present eFORGE signal (average n = 527) compared to those studies that did not (average n = 191). Taken together, these results suggest that tissue-specific enrichment patterns are widespread among EWAS and that eFORGE demonstrates the capacity to detect these patterns.

**Figure 5:**
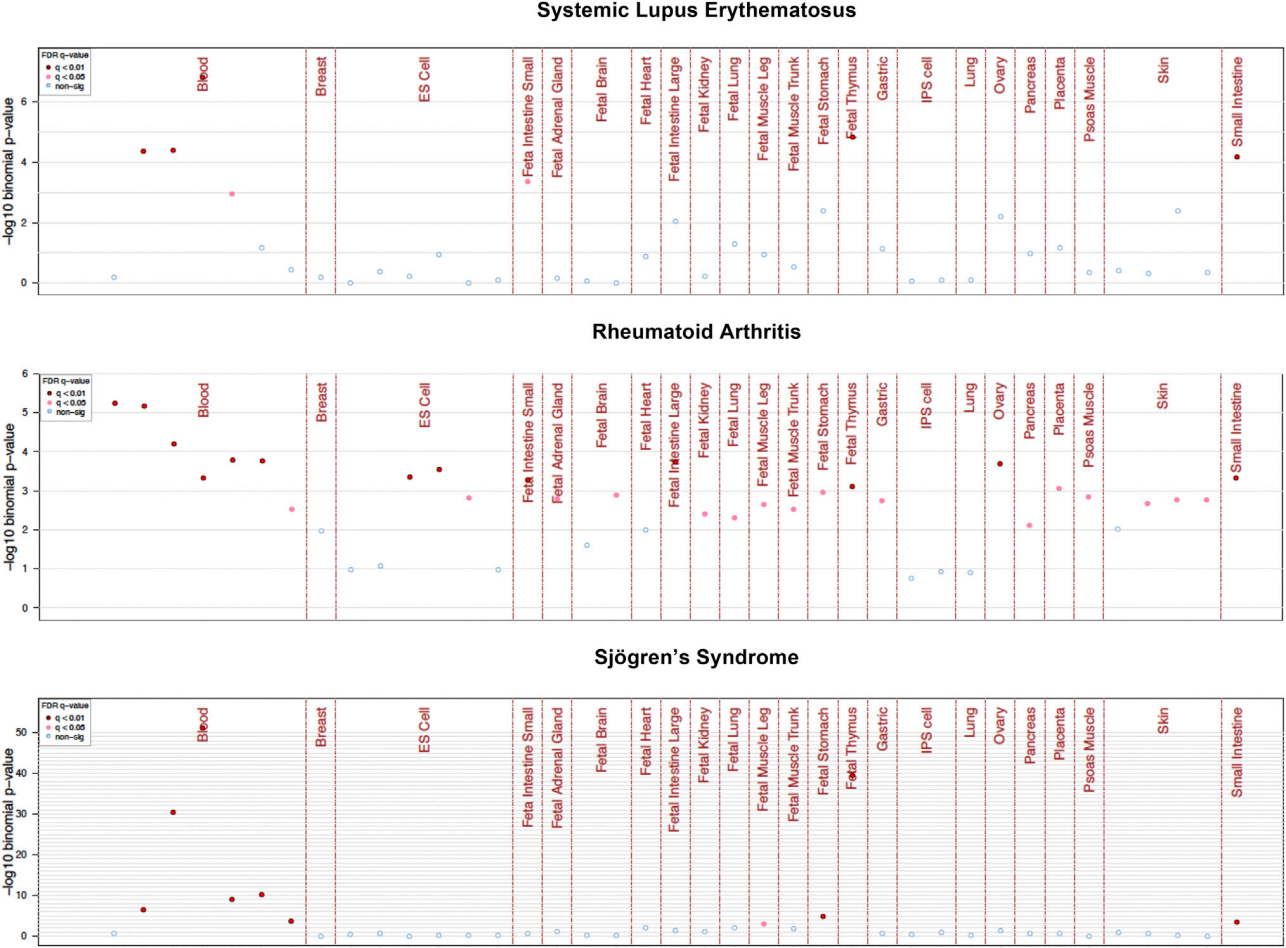
eFORGE Analysis of Autoimmune EWAS Top panel shows a blood (predominantly T cell), intestine, and thymus-specific signal for 86 probes from an EWAS on SLE. Middle panel shows a more general pattern of enrichment, with a strong blood signal, with CD14^+^ cells as the highest category, for a set of 100 RA EWAS probes. Bottom panel shows a blood (predominantly T cell) and thymus-specific enrichment for a set of 753 probes for an EWAS on Sjögren’s syndrome. Probe lists were obtained from the supplementary files of the studies (Coit et al., 2013, Liu et al., 2013, Altorok et al., 2014).

Second, to provide specific examples of eFORGE analysis, we assessed the ability of eFORGE to predict disease-relevant cell types from EWAS conducted on immune blood cells for three autoimmune diseases: rheumatoid arthritis (RA) (Liu et al., 2013), systemic lupus erythematosus (SLE) (Coit et al., 2013), and Sjögren’s syndrome (Altorok et al., 2014). For these diseases, it is assumed that blood is the main affected tissue. We performed eFORGE analyses for each of these diseases using the reported top 100, 86, and 753 probes and consistently found tissue-specific enrichment for immune effector cells and thymus (Figure 5). Specifically, eFORGE results for the EWAS on RA pointed to CD14^+^ cells as the most highly enriched cell type (q value = 5.53e-04), indicating a tendency for cell-composition-corrected RA-associated DNAm changes to co-locate with CD14^+^ DHSs. Indeed, the accelerated maturation of CD14^+^ cells is a hallmark in RA (Hirohata et al., 1996). For the EWAS on SLE, we observed confirmatory enrichment in DHSs specific to T cells (q value = 2.56e-05). T cells, in particular CD4^+^ T cells, play an essential role in the development of SLE (Yin et al., 2015) and have previously been proposed as targets for the treatment of Sjögren’s syndrome (Singh and Cohen, 2012). Consistent with these findings, eFORGE also pointed to an independent T cell signal for DMPs identified in the Sjögren’s syndrome EWAS (q value = 1.31e-49).

**Figure 6:**
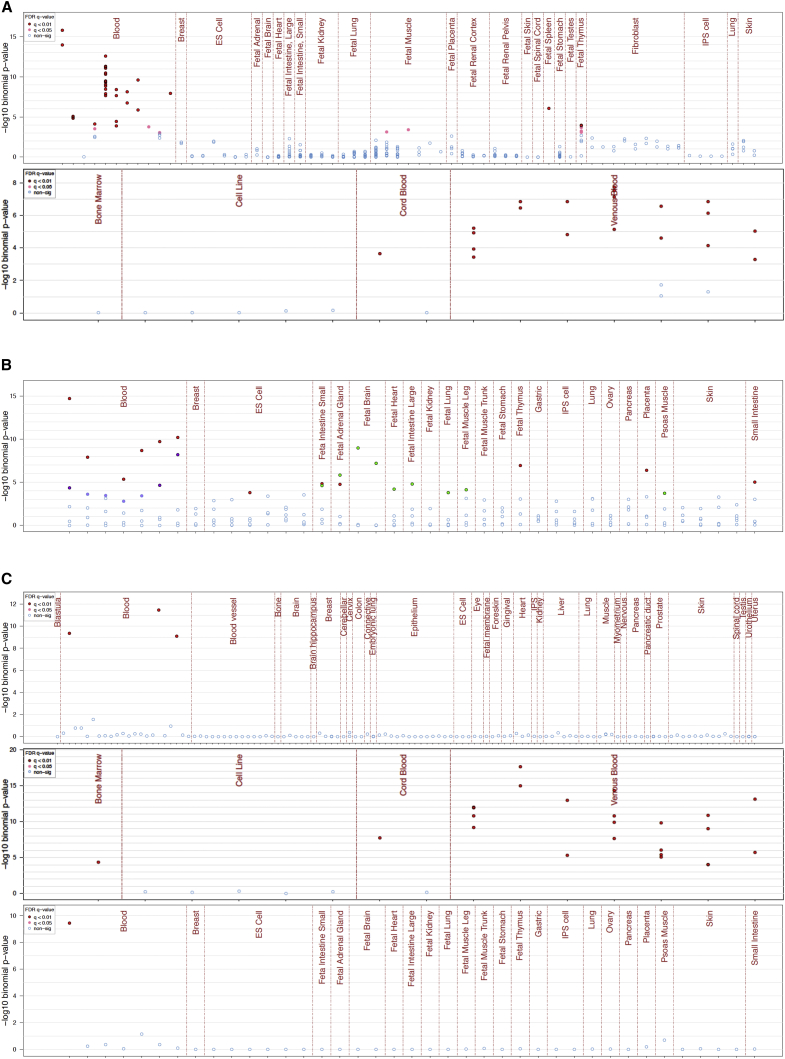
eFORGE Analysis of Surrogate Tissue and Multiple Sclerosis EWAS (A) DHS analysis of multiple sclerosis EWAS. Upper panel shows eFORGE blood, spleen, and thymus enrichment in Roadmap Epigenomics data for top 1,000 hypomethylated DMPs (ranked in the study by likelihood ratio test and Fisher’s method FDR q value). Lower panel shows enrichment for macrophages and monocytes in an analysis of the same regions with BLUEPRINT data. (B) Histone mark analysis of multiple sclerosis EWAS. Panel shows enrichment for top 1,000 study hypomethylated DMRs. Cell type-specific scores are colored by FDR q value. Cell types with q values below 0.01 for histone modifications representative of enhancers (H3K4me1) are shown in red, promoters (H3K4me3) are shown in purple, and polycomb-repressed regions (H3K27me3) are shown in green. Cell types with q values between 0.01 and 0.05 for the histone modification representative of promoters (H3K4me3) are shown in light purple. Cell types with q values above 0.01 for all other histone modifications are shown in blue. H3K36me3 (transcribed regions) and H3K9me3 (a marker for heterochromatin) did not present any significant cell type-specific enrichment patterns. Analyzed regions show enrichment for H3K4me1 (and, at a lower level, H3K4me3) in blood cells. (C) Analysis of surrogate tissue EWAS: the three panels (ENCODE, BLUEPRINT, and consolidated Roadmap, from top to bottom) show enrichment for monocyte, macrophage, and AML for an ovarian cancer prediction EWAS measured on whole blood. There is no enrichment for any other tissue (including lymphoid cells, ovarian tissue, and, interestingly, megakaryocytes). This supports a myeloid-lineage-specific DHS enrichment for top regions from this EWAS. By discarding enrichment in megakaryocyte regions, and showing enrichment for acute promyelocytic leukemia cell lines (NB4 and HL-60), the lineage-specific component of this tissue-specific signal points to a divergence that occurs after differentiation from the common myeloid progenitor and is suggestive of an event during myeloblastic differentiation. This DHS enrichment pattern extends to the myeloblast branch of the myeloid lineage, pointing to these regions being active in the myeloblast, which would be the cell of origin of this tissue-specific signal. This enrichment pattern shows cell types that drive the proposed myeloid/lymphoid imbalance causing the methylation signal observed (Teschendorff et al., 2009, Houseman et al., 2012, Li et al., 2014).

Third, we assessed whether eFORGE can uncover patterns in published EWAS data that would inform the functional interpretation of the statistical findings. Using the top 1,000 hypomethylated regions for an EWAS on multiple sclerosis (MS) (Huynh et al., 2014), we generated eFORGE plots for several DHS reference sets (Figure 6A). We observed a tissue-specific enrichment in immune cells, which is unexpected for a study performed on pathology-free brain tissue. To support this observation, we carried out additional eFORGE analysis using histone marks (Kundaje et al., 2015) that showed an enhancer-specific signature (H3K4me1) underlying this DHS enrichment (Figure 6B). We then intersected the top 1,235 hypomethylated regions from the study that gave rise to the observed immune signal with the locations of active enhancers (n = 1,158) previously identified in microglial cells (Lavin et al., 2014). These immune cells constitute up to 15% of all cells in the mammalian CNS (Xavier et al., 2014). A Fisher’s exact test confirmed significant co-localization of the microglial-specific active enhancers (p value: 2.70e-07, odds ratio [OR]: 5.88, 95% confidence interval [CI]: 3.19–9.96), suggesting that microglial enhancers may be potential drivers of the MS EWAS signal. In conclusion, eFORGE analysis of published MS EWAS data uncovered tissue-specific patterns, suggesting potential molecular mechanisms relevant to the etiology of the disease.

Fourth, we examined whether eFORGE can be used for the interpretation of EWAS that use surrogate tissues. In these studies, DNAm changes are measured in easily accessible tissues such as whole blood or buccal cells rather than the target tissue that is most relevant to the disease. It has been suggested that DNAm changes in surrogate tissues reflect epigenomic perturbations found in the target tissue (Lowe and Rakyan, 2014). An alternative possibility is that the observed epigenetic signature does not mimic methylation changes in the target tissue but is specific to the surrogate tissue. We performed eFORGE analysis on the top 110 regions from an EWAS on ovarian cancer, which was performed on whole blood using a pre-treatment discovery cohort. We found enrichment for CD14^+^ cells (q value = 1.37e-12, Figure 6C), but not for ovary (q value = 1) or solid cancer tissues (q value = 1). In addition, the observed myeloid/lymphoid enrichment patterns suggested cell-composition effects, as only myeloid regions showed enrichment, raising the possibility that these DNAm differences were caused by an increase in myeloid cell numbers in one of the groups. While a similar immune signature had been identified before, in addition to cell-composition effects (Teschendorff et al., 2009, Houseman et al., 2012, Li et al., 2014), we used an alternative approach to support this finding. Specifically, we were able to exclude ovarian signal and provide evidence for which myeloid cell types may drive the EWAS signal. For example, DHS enrichment was not observed for megakaryocytes (Figure 6C). In summary, eFORGE analysis of the top hits from an EWAS on ovarian cancer pointed to cell-composition effects driven by a myeloid-specific immune response to this cancer type, rather than an epigenetic change in peripheral blood that mimics a change in the methylome of the ovarian cancer tissue.

**Figure 7:**
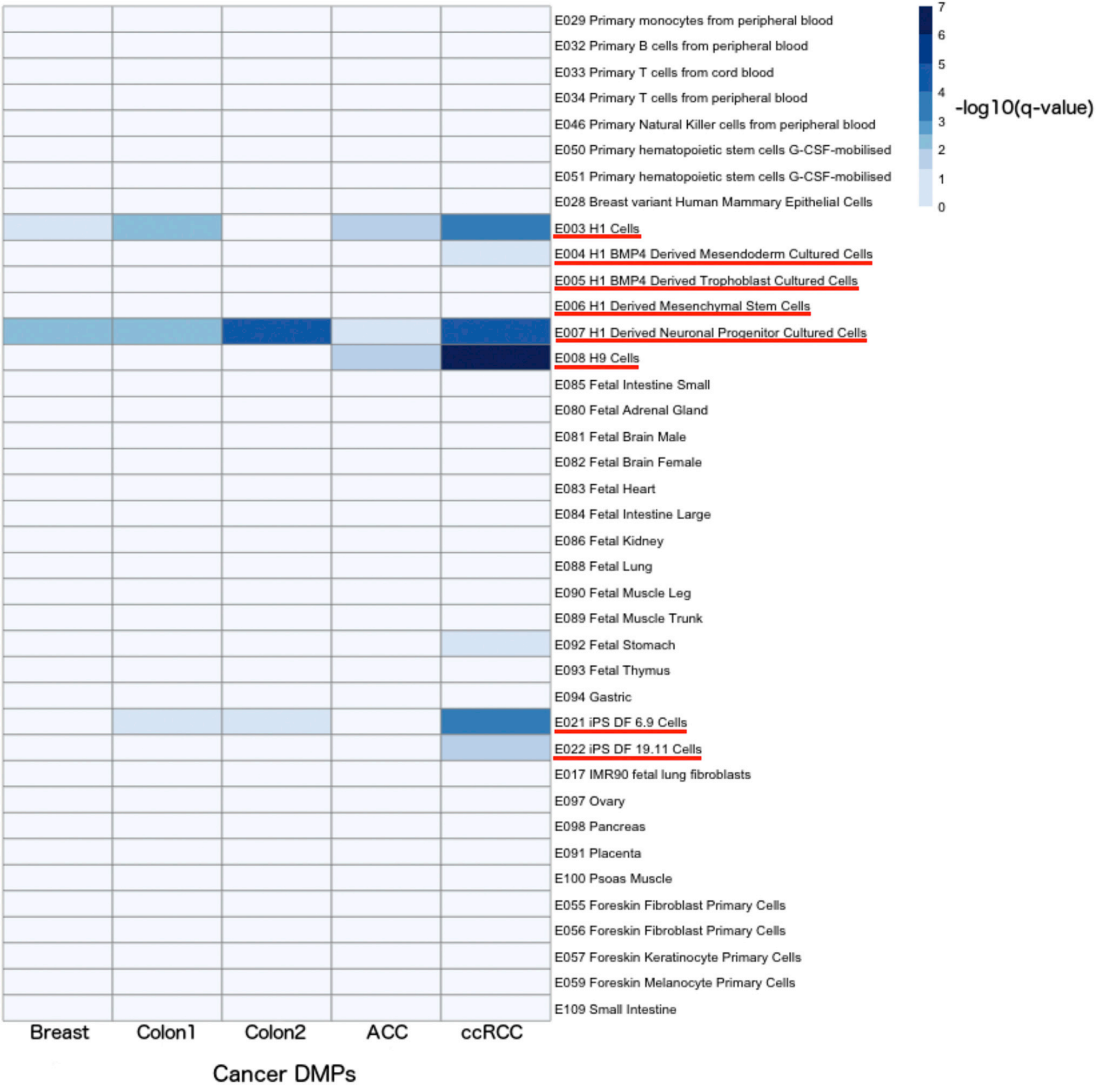
Analysis of Cancer EWAS This heatmap shows a stem cell-like signature for regions from five cancer EWAS, through color-coded enrichment –log10(q value). The left column depicts results for 330 top probes from a breast cancer metastatic behavior EWAS (Fang et al., 2011), the second column from the left shows results for 450 probes from a colorectal carcinoma EWAS (Kibriya et al., 2011), and the central column shows results for 240 probes from a sporadic colorectal cancer EWAS (Laczmanska et al., 2013). The next column on the right shows results for 801 probes from an adrenocortical carcinoma EWAS (Barreau et al., 2013), and the last column on the right shows results for 362 probes from a clear cell renal cell carcinoma EWAS (Arai et al., 2012). All five studies showed intermediate enrichment (q value <0.05) of at least one eFORGE “ES cell” or “iPSC” category. Aside from this stem cell-like signature, no other shared tissue category is enriched across the five probe lists.

Finally, we showed that eFORGE detects tissue-specific patterns in cancer EWAS data. We analyzed five cancer EWAS: breast cancer (Fang et al., 2011), colorectal cancer (Kibriya et al., 2011), sporadic colorectal cancer (Laczmanska et al., 2013), clear cell renal cell carcinoma (Arai et al., 2012), and adrenocortical carcinoma (Barreau et al., 2013). Using the top 330, 450, 240, 801, and 362 EWAS hits, respectively, we performed eFORGE analysis for each DMP set, and identified enrichment in stem cells but not in breast, intestine, or renal tissues across the five studies (Figure 7). This suggests that many regulatory elements affected by cancer epigenetic reprogramming may be stem cell like. This is consistent with previous findings that DNAm changes in cancer tissue aid the emergence of a possible stem cell phenotype (Widschwendter et al., 2007). In conclusion, application of eFORGE to cancer EWAS data provided evidence for a stem cell-like enrichment across all five studies, which warrants further investigation.

## Discussion

We have developed eFORGE, a tool that highlights DMPs identified through EWAS that are likely to be functional in a cell type- and tissue-specific context. Its development represents an addition to the currently limited toolbox available for comprehensive analysis and interpretation of EWAS data. Both the standalone and web-based versions of eFORGE have been subjected to rigorous performance assessment with regards to false-positive rates, reproducibility, and speed to ensure ability to cope with high user demand.

SNPs identified through GWAS have been systematically probed for enrichment at regulatory elements marked by DHSs (Maurano et al., 2012). This analytical approach has recently been implemented in the FORGE tool, which enables an automated analysis workflow (Dunham et al., 2015). Complementing FORGE for epigenetic analysis, we have developed eFORGE to provide large-scale, tissue-specific DHS enrichment analysis for DMPs identified through EWAS. While using parts of the FORGE framework (Dunham et al., 2015), eFORGE uses a different, EWAS-specific background and contains several features not included in FORGE, such as a faster scaling of the binomial test and histone marks as a dataset additional to DHSs.

The main applications of eFORGE are the analysis of EWAS data to predict disease-relevant cell types and potential cell-composition effects, as well as quality-control analysis for studies on tissue-specific DNA methylation. We have provided evidence that eFORGE analysis of tDMPs correctly predicts the relevant tissue through tissue-specific DHS enrichment patterns (Figure 2). In this way, sets of tDMPs can be linked to the corresponding tissue in an independent manner. This link can also be used in the inverse sense to detect regions with a potential tissue-specific regulatory function, by using algorithms to detect probe sets with a high tissue-specific eFORGE score. eFORGE is also designed to aid downstream functional follow-up of DMPs by providing tissue-specific DHS overlap results in the form of tables, with data for specific genomic regions. DHSs are markers of *cis*-regulatory elements (Dorschner et al., 2004), and DNAm changes in these regions may be associated with functional consequences (Schübeler, 2015). Such functional links could be confirmed through experimental assays, including chromatin conformation capture techniques and epigenome editing using CRISPR/Cas9 (Köeferle et al., 2015).

eFORGE can also be used to assess cell-composition effects in EWAS, providing a complementary approach to methylation-based tools. When heterogeneous tissues are analyzed in EWAS, a proportion of the observed differential DNAm signal can be due to cell-composition effects (Houseman et al., 2012, Houseman et al., 2016, Paul and Beck, 2014). eFORGE can identify these cell-composition effects by detecting tissue-specific DHS enrichment based on genomic location, not DNAm values. Unlike alternative methods for detecting cell-composition effects (Zou et al., 2014, Houseman et al., 2012, Houseman et al., 2016), eFORGE provides the user-friendly option of web-based analysis.

eFORGE analysis is, however, not without limitations. Despite the fact that eFORGE can detect cell-composition effects, the user must interpret whether a given enrichment is driven by confounding cell-composition effects or by true cell type-specific effects. Based on the data presented here, strong bias toward one cell type for an EWAS performed on heterogeneous tissue is more likely an indication of cell-composition than cell type-specific effects. The need for a more accurate interpretation requires complementary methods for cell-composition detection and correction to be used and also highlights the requirement of experimental validation. In addition, eFORGE can only analyze cell types for which DHS data are available. Although the current eFORGE database contains 454 samples, many cell types are still not represented. To consider these, we have to recur to alternative analyses, as shown in our analysis of microglial enhancer enrichment outside of eFORGE.

In conclusion, we anticipate eFORGE to contribute to the challenging task ahead of translating the increasing number of DMPs identified through EWAS into relevant molecular mechanisms.

## Experimental Procedures

### DNase I Data

DNase I data from the ENCODE, Roadmap Epigenomics, and BLUEPRINT projects were downloaded and, if necessary, processed using the Hotspot method (see Supplemental Experimental Procedures). Fastq and BAM files for BLUEPRINT samples (listed in Table S6) are available at the European Genome-phenome Archive under accession number EGAD00001002713.

### EWAS DMP Data

A list of 44 450k- and 27k-based EWAS was analyzed with eFORGE. The list of 20 studies with eFORGE signal is contained in Table S7. Studies were selected from Table S1 of a review on EWAS (Michels et al., 2013), taking only studies with a number of samples equal to or above 100. eFORGE analysis of DMP sets was performed for DNase I hotspots from ENCODE, BLUEPRINT, and Roadmap Epigenome projects, with background DMP sets from the Illumina 450k or 27k array where appropriate. For the 27k array background, only probes shared with the 450k array were used. Notably, 13 of the EWAS selected from the aforementioned review did not report top probes. This lack of reporting top probe IDs is an important and not previously reported finding, constituting a major limitation for EWAS reproducibility, in addition to hindering eFORGE analysis of published studies. We urge the community to embrace the open and clear reporting of EWAS results, including top study hits.

### Preparation of eFORGE Overlaps

An SQLite database (http://www.sqlite.org) containing the overlaps for the Infinium Illumina 450k array (Bibikova et al., 2011) cg probes with the BLUEPRINT, ENCODE, and Roadmap Epigenome DNase I hotspots was incorporated into the eFORGE tool. The HumanMethylation450 v.1.2 Manifest File with data from all the cg probes on the 450k array was used to prepare this database (https://support.illumina.com/array/array_kits/infinium_humanmethylation450_beadchip_kit/downloads.html).

We compared the 450k array cg probe data to the indexed DNase I hotspots using the *bedtools* tool (Quinlan and Hall, 2010). The overlaps for each cg probe were stored in an SQLite DB, organized by datasets. For each cg probe, a binary string was generated, representing the presence or absence of an overlap with a hotspot in each dataset (either Roadmap, BLUEPRINT, or ENCODE).

#### Background Probe Parameters

eFORGE evaluated the input DMP set by comparing it to 1,000 background DMP sets. The probes in the background sets were matched to the probes in the input set using annotation (i.e., if one probe from the input set was in a promoter, then its matching probe in each of the 1,000 background sets was also in a promoter). The annotation categories used for this matching process were “Gene annotation” (i.e., 1stExon, 3′ UTR, 5′ UTR, Body, IGR, TSS1500, TSS200) and CpG Island annotation (i.e., Island, Shore_Shelf, and NA or “open sea”). All possible combinations of these two levels resulted in 21 different background bin categories. The CpG island annotation categories “Shelf” and “Shore” were merged to produce enough probes in each background bin category; i.e., more than 1,000. The 450k array Illumina annotation (Bibikova et al., 2011) was used to create these data categories (Tables S1 and S2).

### eFORGE Analysis

Input of DMPs into eFORGE can be in any of two forms: as Illumina 450k/27k probe IDs or as BED format (BED format should be zero based and the chromosome should be given as chrN, as genomic location on human genome assembly GRCh37). Genome coordinates are sufficient to identify probe IDs if these are not provided in BED format. We suggest a minimum of 20 and a maximum of 1,000 probes. If a DMP is not present on the 450k array (or the 27k array probes shared with the 450k array), it is excluded from the analysis. We added a 1-kb proximity filter in order to avoid the biases of testing groups of proximal probes in eFORGE: methylation correlation among closely located CpGs could mean we would be testing the same change more than once. Probes from input are selected at random by the filter, and any probe within 1 kb of any already selected probe is excluded. The choice of selecting 1 kb as a limit for filtering was based on previous data showing strong correlation of DNA methylation levels between CpGs fewer than 1 kb apart (Eckhardt et al., 2006).

Overlaps are retrieved from the eFORGE database for each analyzable probe in the input set. The tool records a count of total hotspot overlaps for each DNase I sample (cell) for the test probe set. eFORGE selects 1,000 matching background probe sets that contain an equal number of probes to the test probe set, matching for gene annotation and CpG island annotation as described above. Retrieval of overlaps from the database for each of the probes in each of the background probe sets then occurs. The tool records an overlap count for each background set in each DNase I sample. For each test probe set, eFORGE obtains the binomial p value for the test set overlap count. This binomial p value is calculated for the test set overlap count relative to the total number of tested probe sets. The binomial test was chosen over the hypergeometric test due to the important computational speed advantages it offers, which are further highlighted considering the high number of tests performed by eFORGE.

### eFORGE Outputs

Tabular and graphic descriptions of the enrichment of overlap for the test DMPs are generated by eFORGE for each DNase I hotspot sample. The tool outputs a tab-separated values (TSVs) file, which includes columns for *Z* score, binomial p value, cell, tissue, datatype, filename of the sample hotspots, DMPs that contribute to the enrichment, the GEO accession for each sample, and the BY adjusted q value. An interactive table containing these data is generated using the Datatables (https://datatables.net/) plug-in for the jQuery JavaScript library accessed through the rCharts package (http://ramnathv.github.io/rCharts/).

In terms of graphical visualization of eFORGE output, FORGE scripts (Dunham et al., 2015) were adapted with minor modifications. Briefly, the –log 10 (binomial p value) is presented by cell sample in each of the graphic outputs. Base R graphics (http://www.r-project.org) are used to generate a pdf graphic. eFORGE generates the interactive JavaScript graphic using the rCharts package (http://ramnathv.github.io/rCharts/) to interface with the dimple d3 libraries (http://dimplejs.org). In the pdf and the interactive graphic cells are grouped alphabetically within each tissue (tissues, in turn, also follow alphabetical order). The coloring of results by BY-corrected q value in each of the graphics is consistent, blue (q value >0.05), pink (q value <0.05), and red (q value <0.01).

### eFORGE Reproducibility

A default probe set of 11 monocyte-specific DMPs was run 1,000 times on eFORGE, and we plotted a resulting longitudinal set of CD14^+^ category q values for these runs (Figure 1B).

### False-Positive Testing

We tested eFORGE BY correction with sets of 5, 10, 15, 20, 30, 40, 50, and 100 randomly selected probes, performing 5,000 analyses for both Roadmap Epigenomics and ENCODE data. We did not obtain any false-positives (at a q value <0.01) for any of these analyses. We did, however, obtain false-positives at a q value <0.05 (0.36%), but this is only indicated to be an intermediate level of significance in eFORGE. The false-positive rates at a q value <0.05 do not show a clear tendency with the size of the probe sets (see Tables S3 and S4). Most of the q values below 0.05 come from a few probe sets, signaling that most FP come from random sets that are borderline for many tissues/cell lines. This supports the notion that it is highly improbable to obtain eFORGE tissue-specific enrichment with random probe lists.

#### Comparison of BH and BY

8,000 sets ranging between five and 100 DMPs were run on eFORGE using both BH and BY multiple testing correction methods. As 299 samples were analyzed for each of the 8,000 DMP sets, there is a total number of 2,392,000 sample tests. Out of these random tests, one false-positive was recorded for BH, and zero were recorded for BY, at a significance level of 0.01.

#### Addition of New Data to eFORGE Database

All the code for generating the eFORGE database from scratch is openly available on GitHub (see https://github.com/charlesbreeze/eFORGE/blob/master/database/README.txt and examples in the same directory). Data from a new project can be added as a table to the modular SQLite database. In addition, indications are provided for the production of bitstring tables from raw data.

#### GeEC Analysis

Datasets used for GeEC correlation analysis were taken from the IHEC Data Portal (http://epigenomesportal.ca/ihec/) as of March 2016. They correspond to 508 DNase sequencing (DNase-seq) datasets generated by ENCODE, Roadmap, and BLUEPRINT, as well as 1,277 chromatin immunoprecipitation sequencing (ChIP-seq) datasets for five core histone marks (H3K4me1, H3K4me3, H3K36me3, H3K27me3, and H3K9me3) generated by ENCODE and Roadmap. These 1,785 datasets were processed with the Genomic Efficient Correlator (GeEC) tool to first average the signal of each dataset in non-overlapping bins of 10 kb (excluding the ENCODE blacklisted regions) and then to calculate a matrix of the pairwise Pearson product-moment correlation coefficients (r). Hierarchical clustering (using the 1-r distance metric and the average linkage method) was then conducted, and the principal clusters were annotated.

Adjusted Rand Index (ARI) results were also used to validate the accuracy of the clustering by using the IHEC-provided metadata describing every dataset for three categories of labels (assayType, cellType, consortium). The tree generated through hierarchical clustering is cut at the appropriate height to obtain a number of clusters equal to the number of distinct labels in each category and then used to calculate the ARI scores. ARI values were calculated using the “average” linkage method.

### tDMP Analysis

We extracted normalized samples from Marmal-aid (Lowe and Rakyan, 2013) for tissues that contained a minimum of 50 samples. We used the dmpFinder function in minfi (Aryee et al., 2014) to make an initial call of tissue-specific methylation differences. Two categories were defined to perform this for each tissue: group 1 contained the tissue of interest, and other tissues were included in group 2. We used the top two probes for each of the tissue-specific calls to visually inspect the data. Any samples that were found to be closer to the mean of group 2 than group 1 were removed. The final calls were then produced using the remaining samples. We randomly selected 50 samples for each tissue, and we then followed a similar procedure to the above, calling differences using dmpFinder. Data S2 contains the IDs of the samples used in the analysis. Data S3, S4, and S5 contain the calls for three of the tissues for all probes.

### cDMP Analysis

We obtained data from Table S2 of a cell type-specific methylation paper by Jaffe and Irizarry (2014). cDMP selection was based on a given probe having the lowest methylation value for a given cell type when compared to the other cell types, and this low methylation value being lower than the next closest methylation value by 0.4.

### Source Code

The source code for eFORGE is available on GitHub at https://github.com/charlesbreeze/eForge. It includes the code for the standalone tool, the webserver, and the scripts to build or extend the database, although a pre-compiled eForge.db sqlite database and background selection hash tables are readily available at http://eforge.cs.ucl.ac.uk/?download. Code variants (e.g., probe weighting) are discussed in the Supplemental Experimental Procedures. eFORGE has been successfully installed and run on Red Hat Linux and OS X 10.9.5.

## Author Contributions

C.E.B., D.S.P., I.D., E.B., J.H., and S.B. designed the project. J.v.D., L.M.B., R.L., V.K.R., M.F., K.D., W.H.O., E.V., S.S., F.M., A.K.B., R.S., J.L., P.-E.J., GB, J.H.A.M., and H.G.S. provided data. C.E.B., I.D., and J.H. designed and developed the software package with support from A.E.T., J.C.A., J.E.B., and V.I. C.E.B., S.B., and D.S.P. wrote the paper with contributions from all authors.

## Accession Numbers

The accession number for the BLUEPRINT data reported in this paper is EGA: EGAD00001002713.

## Supplemental Information

Document S1. Supplemental Experimental Procedures, Figures S1–S7, Tables S1–S5, and S7

Table S6. eFORGE BLUEPRINT Sample Data, Related to Figure 1

Data S1. eFORGE Datasets, Related to Figure 1

Data S2. tDMP Datasets, Related to Figure 2

Data S3. Blood tDMPs, Related to Figure 2

Data S4. Kidney tDMPs, Related to Figure 2

Data S5. Lung tDMPs, Related to Figure 2

Document S2. Article plus Supplemental Information
