# Supplementary material for: eFORGE: A Tool for Identifying Cell Type-Specific Signal in Epigenomic Data
Source: Cell Rep. 2016 Nov 15;17(8):2137–50. doi: 10.1016/j.celrep.2016.10.059 (PMC5120369; doi:10.1016/j.celrep.2016.10.059)
Supplement: Document S1. Supplemental Experimental Procedures, Figures S1–S7, Tables S1–S5, and S7 [file mmc1.pdf]

Cell Reports, Volume *17*

Supplemental Information

eFORGE: A Tool for Identifying

Cell Type-Specific Signal in Epigenomic Data

Charles E. Breeze, Dirk S. Paul, Jenny van Dongen, Lee M. Butcher, John C. Ambrose, James E. Barrett, Robert Lowe, Vardhman K. Rakyan, Valentina Iotchkova, Mattia Frontini, Kate Downes, Willem H. Ouwehand, Jonathan Laperle, Pierre-Étienne Jacques, Guillaume Bourque, Anke K. Bergmann, Reiner Siebert, Edo Vellenga, Sadia Saeed, Filomena Matarese, Joost H.A. Martens, Hendrik G. Stunnenberg, Andrew E. Teschendorff, Javier Herrero, Ewan Birney, Ian Dunham, and Stephan Beck

A

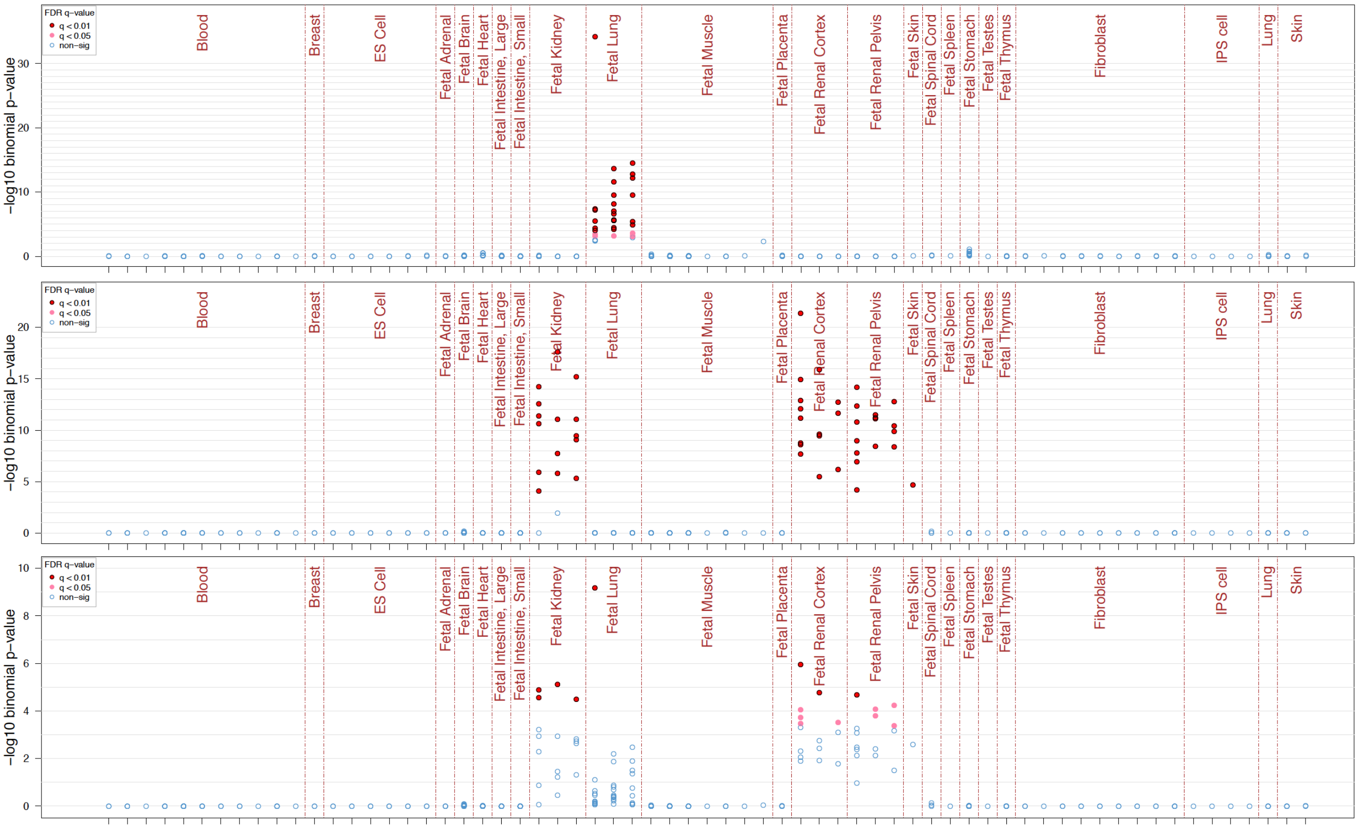
FDR q-value
q < 0.01
q < 0.05
non-sig
−log10 binomial p-value
Blood
Breast
ES Cell
Fetal Adrenal
Fetal Brain
Fetal Heart
Fetal Intestine, Large
Fetal Intestine, Small
Fetal Kidney
Fetal Lung
Fetal Muscle
Fetal Placenta
Fetal Renal Cortex
Fetal Renal Pelvis
Fetal Skin
Fetal Spinal Cord
Fetal Spleen
Fetal Stomach
Fetal Testes
Fetal Thymus
Fibroblast
IPS cell
Lung
Skin

B

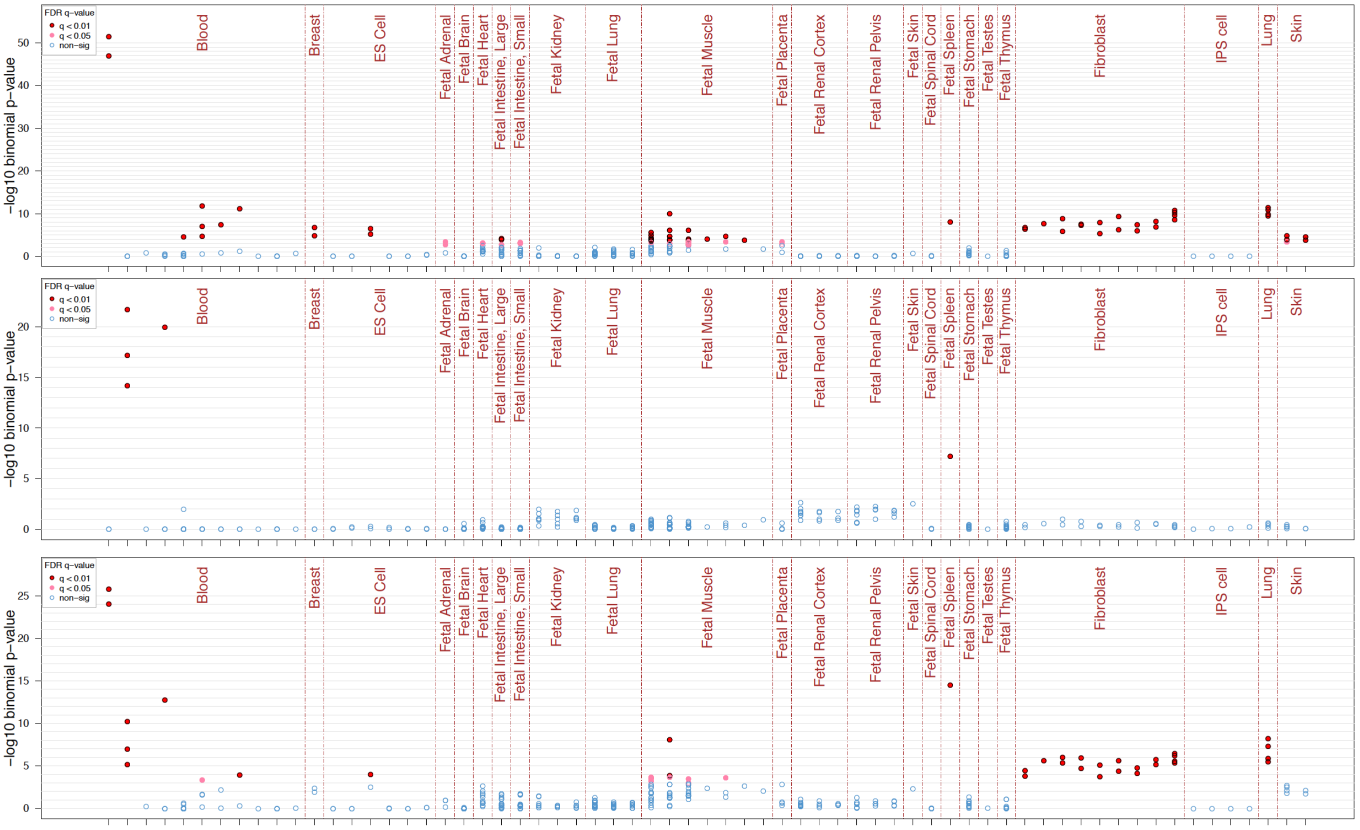
FDR q-value
q < 0.01
q < 0.05
non-sig
−log10 binomial p-value
Blood
Breast
ES Cell
Fetal Adrenal
Fetal Brain
Fetal Heart
Fetal Intestine, Large
Fetal Intestine, Small
Fetal Kidney
Fetal Lung
Fetal Muscle
Fetal Placenta
Fetal Renal Cortex
Fetal Renal Pelvis
Fetal Skin
Fetal Spinal Cord
Fetal Spleen
Fetal Stomach
Fetal Testes
Fetal Thymus
Fibroblast
IPS cell
Lung
Skin

Figure S1. eFORGE analysis of mixed DHS overlapping tDMP and cDMP sets. Related to Figure 2. [A]: eFORGE analysis of a mixed set of DHS-overlapping tDMPs. The top panel shows enrichment in lung for a list of 55 DHS-overlapping lung tDMPs. The middle panel shows enrichment in kidney for a set of 51 DHS-overlapping kidney tDMPs. The bottom panel shows mixed tissue-specific enrichment for lung and kidney for the merged list of 106 probes. **[B]:** eFORGE analysis of a mixed set of DHS-overlapping cDMPs. The top panel shows enrichment results from testing 148 monocyte specific DMPs, highlighting the CD14+ category. The middle panel shows enrichment results for 148 B cell specific DMPs, highlighting mainly the CD19+ and spleen categories. The bottom panel shows enrichment results for a mixed set of both cell type-specific probe sets. As evident from the mixed set, enrichments for both cell types are still present.

Intergenic region	Island	Shore_Shelf	NA
Within the 200 bp prior to a Transcription start site	Island	Shore_Shelf	NA
1st Exon	Island	Shore_Shelf	NA
Within the 1500 bp prior to a Transcription start site	Island	Shore_Shelf	NA
3' Untranslated region	Island	Shore_Shelf	NA
5' Untranslated region	Island	Shore_Shelf	NA
Gene Body	Island	Shore_Shelf	NA

Table S1. Data categories for background probe matching in eFORGE. Related to Figure 1. Column 1 indicates gene-centric category and columns 2, 3 and 4 indicate the three subsequent CpG Island-centric subcategories that separate probes from each gene-centric category.

IGR	Shore	21121
IGR	Island	22392
IGR	NA	57749
IGR	Shelf	18390
TSS200	Shore	9372
TSS200	Island	32996
TSS200	Shelf	857
TSS200	NA	9058
1stExon	Shore	2506
1stExon	Island	15581
1stExon	Shelf	368
1stExon	NA	4282
TSS1500	Shore	31022
TSS1500	Island	21610
TSS1500	Shelf	1685
TSS1500	NA	14667
3'UTR	Shore	3426
3'UTR	Island	1992
3'UTR	Shelf	1802
3'UTR	NA	10274
5'UTR	Shore	9460
5'UTR	Island	17581
5'UTR	NA	11855
5'UTR	Shelf	3789
Body	Shore	35160
Body	Island	38102
Body	Shelf	20253
Body	NA	68162
	Total	485512

Table S2. Numbers of probes in each background bin category in eFORGE. Related to Figure 1. The selection of each background probe matching each DMP in the test set is based on gene-centric and CpG island-centric annotation.

SetSize	N	p<0.05	p<0.01	q<0.05	q<0.01	F	p1f	p2f	q1f	q2f
5	625000	2418	190	0	0	5000	936	104	0	0
10	625000	23686	3602	23	0	5000	2829	943	11	0
15	625000	5721	597	0	0	5000	1338	283	0	0
20	625000	9313	1226	32	0	5000	1887	426	3	0
30	625000	13487	1591	4	0	5000	2274	542	1	0
40	625000	10877	1356	6	0	5000	1999	475	5	0
50	625000	13714	1554	16	0	5000	2222	570	4	0
100	625000	12973	1593	1	0	5000	2229	554	1	0

Table S3. Results from testing 5000 random probe sets for false positives on ENCODE data. Related to Figure 1. At q<0.01 we do not observe any false positives for any input test size (ranging between 5 and 100 probes).

SetSize	N	p<0.05	p<0.01	q<0.05	q<0.01	F	p1f	p2f	q1f	q2f
5	1495000	4161	313	0	0	5000	1013	142	0	0
10	1495000	54869	7930	264	0	5000	2998	1065	6	0
15	1495000	10519	904	0	0	5000	1544	316	0	0
20	1495000	16263	1570	1	0	5000	1894	431	1	0
30	1495000	28526	3279	0	0	5000	2214	602	0	0
40	1495000	22855	2557	129	0	5000	1997	496	2	0
50	1495000	30460	3430	1	0	5000	2341	672	1	0
100	1495000	23173	2480	1	0	5000	2051	510	1	0

Table S4. Results from testing 5000 random probe sets for false positives on Roadmap Epigenomics data. Related to Figure 1. At q<0.01 we do not observe any false positives for any input test size (ranging between 5 and 100 probes).

**Datatype**	**Assay**	**Celltype**	**Consortium**
**All (1785)**	0.49 {6}	0.08 {305}	-0.04 {3}
**H3K36me3 (255)**	1 {1}	0.32 {138}	-0.04 {2}
**H3K4me3 (292)**	1 {1}	0.25 {165}	-0.01 {2}
**H3K4me1 (239)**	1 {1}	0.37 {128}	0.06 {2}
**H3K27me3 (253)**	1 {1}	0.35 {134}	0.03 {2}
**H3K9me3 (238)**	1 {1}	0.26 {126}	0.07 {2}
**DNase-Seq (508)**	1 {1}	0.40 {183}	0.01 {3}

Table S5: Adjusted Rand Index (ARI) results for ENCODE, BLUEPRINT and Roadmap Epigenomics data clustering with GeEC. Related to Figure 1. An ARI score around zero means that the labels of a category are randomly distributed among clusters, while a positive value (max of 1) means that the labels of a category are enriched in a defined cluster. Numbers in ( ) represent the number of datasets in each group of datasets, while numbers in { } represent the number of different labels for each category and group. ARI results show that the contribution of "Consortium" is small, and the main labels for clustering are "Assay" and "Celltype".

Table S6: eFORGE BLUEPRINT sample data. Related to Figure 1. Sample data includes file name, lab of origin, type of data (all samples are DHS), cell name, tissue of origin, short cell name (for plotting), sample individual data (sex, age and disease status), sample URL and URL of original file. Samples include both primary cells and transformed cell lines. This table has been supplied as a Supplemental Excel file.

Study number	Number of samples	PMID	Disease/Trait	Platform	Analysed tissue	eFORGE tissue	eFORGE link
1	2442	23034122	Ageing in blood and brain **;***	27 and 450k	Whole blood, leukocytes, brain	Stem cell signal for 1000 probes from additional file 4	http://eforge.cs.ucl.ac.uk/eFORGE.v1.2/files/0xC8FF623812ED11E6B81D459BF8274B10/additional_file_4_ageing_EWAS.450k.erc2-DHS.chart.pdf
2	135	23093492	Adrenocortical carcinoma**	27k	Adrenocortical carcinoma tumours	Stem cell signal for probes from supplemental table 1 (362 probes) under 0.01	http://eforge.cs.ucl.ac.uk/eFORGE.v1.2/files/0x4D980C8425B311E6B2D23DDCF8274B10/index.html
3	910	23578854	Breast cancer	27k	blood	Mixed enrichment for several tissues for probes from Supplemental Table 1	http://eforge.cs.ucl.ac.uk/eFORGE.v1.2/files/0x439DAF16242011E6B310C6D3F8274B10/No_label_given.450k.erc2-DHS.chart.pdf
4	138	22610075	Clear cell renal cell carcinomas (RCCs)**	27k	29 normal renal cortex tissue, 109 tumor tissues and 107 matched adjacent normal tissue	Stem cell enrichment and other mixed enrichment for probes from supplemental table 2 (801 probes)	http://eforge.cs.ucl.ac.uk/eFORGE.v1.2/files/0x5550140A115211E6AF74B300F9274B10/No_label_given.450k.erc2-DHS.chart.pdf
5	367	21297937	Congenital Heart Defects (CHDs)	27k	blood	Mixed enrichment for probes from Table S1 (top 20 positive probes)	http://eforge.cs.ucl.ac.uk/eFORGE.v1.2/files/0xF3288FF2240A11E6A6E27FB6F8274B10/No_label_given.450k.erc2-DHS.chart.pdf
6	192	20687937	Diabetic nephropathy**	27k	Whole blood	Mixed enrichment for probes from supplemental table s1, with q values below 0.15	http://eforge.cs.ucl.ac.uk/eFORGE.v1.2/files/0x149CA7F4120E11E68EF480F5F8274B10/bell_diabetic_nefropathy_qvals_0_15.450k.erc2-DHS.chart.pdf
7	1230	22714737	Nonhematopoietic cancers and blood leukocyte profiles	27k	purified peripheral blood leukocyte subtypes from healthy donors	CD14 cell signal for probes from Supplemental Table 4	http://eforge.cs.ucl.ac.uk/eFORGE.v1.2/files/0x62FCC88C240D11E6A13229BAF8274B10/No_label_given.450k.erc2-DHS.chart.pdf
8	168	21453505	Parental age**	27k	cord blood	Blood signal for probes from table s5.	http://eforge.cs.ucl.ac.uk/eFORGE.v1.2/files/0x451A5388160D11

							E6945594A5F8274B10/No_label_given.450k.erc2-DHS.chart.pdf
9	201	21308978	Racial differences at birth**	27k	cord blood	Blood signal for "yes" probes from table s4:	http://eforge.cs.ucl.ac.uk/eFORGE.v1.2/files/0xBC47687C161411E6BF88B9AFF8274B10/No_label_given.450k.erc2-DHS.chart.pdf
10	255	23579546	Breast cancer	27k, 450k	breast	Blood and thymus signal for probes from Bre100 Supplemental Table 2A	http://eforge.cs.ucl.ac.uk/eFORGE.v1.2/files/0xC4946164242011E68EC468D4F8274B10/No_label_given.450k.erc2-DHS.chart.pdf
11	317	23526956	Clear cell renal cell carcinoma (ccRCC)	27k, 450k	kidney (Tumour and adjacent non-tumour tissue)	Kidney signal for top 200 methylated 450k probes from table S6	http://eforge.cs.ucl.ac.uk/eFORGE.v1.2/files/0xE35B9F46241F11E693220FD2F8274B10/No_label_given.450k.erc2-DHS.chart.pdf
12	656	23177740	Ageing***	450k	Whole blood	Stem cell signal for probes from table s5 model kidney, the largest TCGA category (183 Kidney samples, compared to 83 breast, 60 lung, and 42 skin samples):	http://eforge.cs.ucl.ac.uk/eFORGE.v1.2/files/0x2B74E1A8137B11E6AF31C3D2F8274B10/No_label_given.450k.erc2-DHS.chart.pdf
13	192	23332324	Child maltreatment***	450k	Salivary DNA	Mixed enrichment with blood skew for top 1000 probes by p-value from annex B, supplemental information	http://eforge.cs.ucl.ac.uk/eFORGE.v1.2/files/0x6B81FA2E12EA11E6A1B50A97F8274B10/appendix_B_child_abuse_EWAS.450k.erc2-DHS.chart.pdf
14	691	23334450	Rheumatoid arthritis	450k	blood	Blood enrichment, with other tissues, for probes from Supplemental Table 3	http://eforge.cs.ucl.ac.uk/eFORGE.v1.2/files/0xA38CF988241E11E68AF555D0F8274B10/No_label_given.450k.erc2-DHS.chart.pdf
15	184	23175441	Smoking status and cancer (breast and colon cancer)	450k	blood	Stomach enrichment for breast cancer comparison data	http://eforge.cs.ucl.ac.uk/eFORGE.v1.2/files/0x0E0FEA7E240E11E6A42C34BBF8274B10/No_label_given.450k.erc2-DHS.chart.pdf

16	1793	23691101	Tobacco smoking***	450k	Whole blood	Blood and thymus as main categories for probes from table s1	http://eforge.cs.ucl.ac.uk/eFORGE.v1.2/files/0x3C0CE156121211E68B61CBFAF8274B10/table_s1_tobacco.450k.erc2-DHS.chart.pdf
17	138	22232023	Tobacco smoking***	450k	119 lymphoblast DNA and 19 lung alveolarmacrophage DNA	Breast epithelial signature potentially signalling macrophage (BP confirmation) for probes from table 5	http://eforge.cs.ucl.ac.uk/eFORGE.v1.2/files/0x81C7A70412D911E687D76CFFF8274B10/No_label_given.450k.erc2-DHS.chart.pdf
18	153	23064414	Two subtypes of CLL; mutated or unmutated IGHV***	450k	Tumor samples and B cells from the controls	Mixed enrichment for top 1000 Met M-CLL / unmet U-CLL probes from supp table 5, with 3 out of 4 highest categories as blood subtypes, and also lung as enriched category	http://eforge.cs.ucl.ac.uk/eFORGE.v1.2/files/0xDA59362C0E2311E6A0BCC9DDF8274B10/index.html
19	261	20019873	Ovarian cancer**	27k	peripheral blood	CD14+ blood signal for a blood based EWAS for top 419 probes from supplemental table 2, agrees with article GSEA analysis results of immune specific genes, this is for 27k analysis	http://eforge.cs.ucl.ac.uk/eFORGE.v1.1/files/0x33752BF89CD311E4BEA3AC33AA596114
20	114	23666970	Thyroid cancer subtypes**	27k	114 snap-frozen thyroid samples	Cross-tissue mixed enrichment for first 1000 probes from supplemental table 2, highest category stem cell	http://eforge.cs.ucl.ac.uk/eFORGE.v1.2/files/0xA8797ECC0E0111E694B84EB2F8274B10/No_label_given.450k.erc2-DHS.chart.pdf

Table S7. List of analysed EWAS. Related to Figure 3 and Figure 4. Study information (columns 1-6), eFORGE enriched cell type (column 7) and eFORGE URL (column 8).

Supplemental files:

File S1. eFORGE datasets. Related to Figure 1.

File S2. tDMP datasets. Related to Figure 2.

File S3. Blood tDMPs. Related to Figure 2.

File S4. Kidney tDMPs. Related to Figure 2.

File S5. Lung tDMPs. Related to Figure 2.

Supplemental Experimental Procedures

DNase I Data

Hotspots from the ENCODE project (Thurman et al., 2012) were taken from ftp://ftp.ebi.ac.uk/pub/databases/ensembl/encode/integration_data_jan2011/byDataType/openchrom/jan2011/combined_hotspots/. Hotspots from the BLUEPRINT project were downloaded from https://blueprint.genomatix.de/grid/experiments/browse (**Table S6**). DNase I sequencing tag alignments from the Roadmap Epigenome Project were taken from http://www.genboree.org/EdaccData/Current-Release/experiment-sample/Chromatin_Accessibility/. The files that were used belong to the section of Gene Expression Omnibus (GEO) accession number GSE18927 available according to the data use embargo agreement. The Hotspot method (http://www.uwencode.org/proj/hotspot/) (John et al., 2011) (Sabo et al., 2004) was used to process the alignments, using the default parameters to give hotspot and peak files. Consolidated Roadmap Epigenomics DNase I hotspots and BroadPeak Histone mark data were retrieved from http://egg2.wustl.edu/roadmap/data/byFileType/peaks/consolidated/broadPeak/. Each of the datasets was assigned to the relevant cells and tissues using either consortium data or custom perl scripts from the decodings which are available from the ENCODE Data Coordination Centre tables (https://genome.ucsc.edu/encode/cellTypes.html) or from the BioSamples database sample group SAMEG31306 (http://www.ebi.ac.uk/biosamples/).

eFORGE probe weighting

Weights can be included in standalone eFORGE as follows. The initial weight can be set with the –weights flag and the step to generate subsequent weights can be set with the –step flag (see documentation at https://github.com/charlesbreeze/eFORGE/blob/eforge.v1.0/eforge.pl). Initial results show that weighting can improve certain enrichments, and better reflect the different significance (or effect size) of probes in the input list. However, we advise that the effect on the underlying statistics should be taken into consideration when introducing weights, and we advise caution when interpreting weighted results.

Supplemental References

John, S., Sabo, P.J., Thurman, R.E., Sung, M.-H., Biddie, S.C., Johnson, T.A., Hager, G.L., and Stamatoyannopoulos, J.A. (2011). Chromatin accessibility pre-determines glucocorticoid receptor binding patterns. Nat. Genet. *43*, 264–268.

Sabo, P.J., Hawrylycz, M., Wallace, J.C., Humbert, R., Yu, M., Shafer, A., Kawamoto, J., Hall, R., Mack, J., Dorschner, M.O., et al. (2004). Discovery of functional noncoding elements by digital analysis of chromatin structure. Proc. Natl. Acad. Sci. U. S. A. *101*, 16837–16842.

Thurman, R.E., Rynes, E., Humbert, R., Vierstra, J., Maurano, M.T., Haugen, E., Sheffield, N.C., Stergachis, A.B., Wang, H., Vernot, B., et al. (2012). The accessible chromatin landscape of the human genome. Nature *489*, 75–82.
