# Supplementary material for: eFORGE: A Tool for Identifying Cell Type-Specific Signal in Epigenomic Data
Source: Cell Rep. 2016 Nov 15;17(8):2137–50. doi: 10.1016/j.celrep.2016.10.059 (PMC5120369; doi:10.1016/j.celrep.2016.10.059)
Supplement: Document S2. Article plus Supplemental Information [file mmc8.pdf]

Cell Reports

Resource

eFORGE: A Tool for Identifying Cell Type-Specific Signal in Epigenomic Data

Graphical Abstract

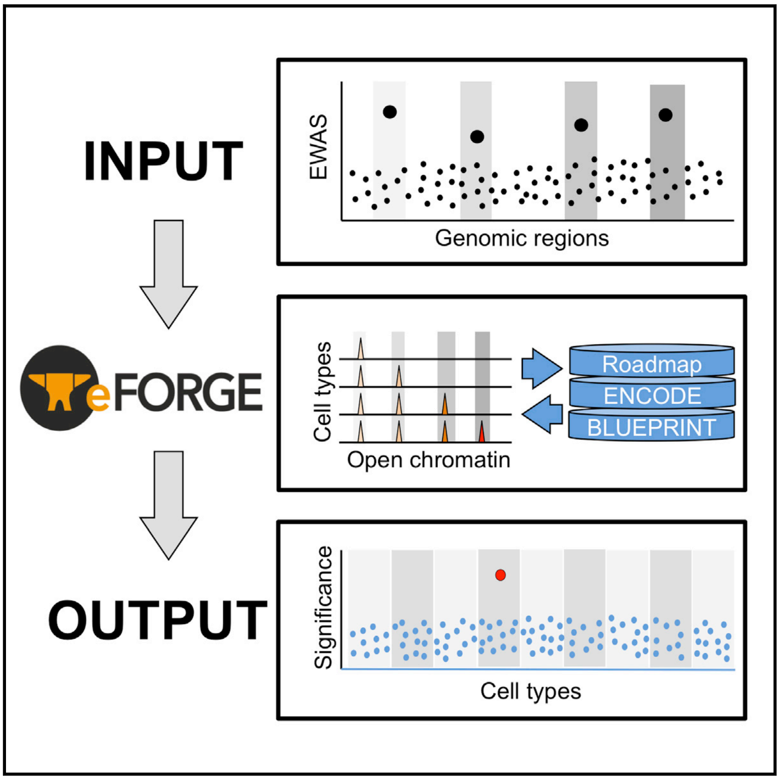

Authors

Charles E. Breeze, Dirk S. Paul, Jenny van Dongen, ..., Ewan Birney, Ian Dunham, Stephan Beck

Correspondence

c.breeze@ucl.ac.uk (C.E.B.),
s.beck@ucl.ac.uk (S.B.)

In Brief

Breeze et al. develop a tool for the analysis and interpretation of EWAS data. The eFORGE tool identifies cell type-specific, disease-relevant signals in heterogeneous EWAS data and can also identify cell-composition effects. Explore consortium data at the Cell Press IHEC webportal at http://www.cell.com/consortium/IHEC.

Highlights

- Development of a tool for the analysis and interpretation of EWAS data
- Identification of cell type-specific signals in heterogeneous EWAS data
- Identification of cell-composition effects in EWAS
- Compilation of eFORGE catalog of 20 published EWAS

Breeze et al., 2016, Cell Reports *17*, 2137–2150
November 15, 2016 © 2016 The Author(s).
http://dx.doi.org/10.1016/j.celrep.2016.10.059

Cell Reports
Resource

OPEN ACCESS
CellPress

eFORGE: A Tool for Identifying Cell Type-Specific Signal in Epigenomic Data

Charles E. Breeze,[1,21,*] Dirk S. Paul,[1] Jenny van Dongen,[2] Lee M. Butcher,[1,3] John C. Ambrose,[1] James E. Barrett,[1] Robert Lowe,[4] Vardhman K. Rakyan,[4] Valentina Iotchkova,[5,6] Mattia Frontini,[7,8,9] Kate Downes,[7,8] Willem H. Ouwehand,[6,7,8,9] Jonathan Laperle,[10] Pierre-Étienne Jacques,[10,11,12] Guillaume Bourque,[13,14] Anke K. Bergmann,[15,16] Reiner Siebert,[15,17] Edo Vellenga,[18] Sadia Saeed,[19,20] Filomena Matarese,[20] Joost H.A. Martens,[20] Hendrik G. Stunnenberg,[20] Andrew E. Teschendorff,[1] Javier Herrero,[1] Ewan Birney,[5] Ian Dunham,[5] and Stephan Beck[1,*]
[1]UCL Cancer Institute, University College London, London WC1E 6BT, UK
[2]Department of Biological Psychology, Vrije Universiteit Amsterdam, 1081BT Amsterdam, the Netherlands
[3]Department of Surgery and Cancer, Imperial College London, London W12 0NN, UK
[4]Blizard Institute, Barts and The London School of Medicine and Dentistry, Queen Mary University of London, E1 2AT London, UK
[5]European Molecular Biology Laboratory, European Bioinformatics Institute (EMBL-EBI), Wellcome Genome Campus, Hinxton, Cambridge CB10 1SD, UK
[6]Department of Human Genetics, The Wellcome Trust Sanger Institute, Wellcome Genome Campus, Hinxton, Cambridge CB10 1HH, UK
[7]Department of Haematology, University of Cambridge, Cambridge Biomedical Campus, Long Road, Cambridge CB2 0PT, UK
[8]National Health Service (NHS) Blood and Transplant, University of Cambridge, Cambridge Biomedical Campus, Long Road, Cambridge CB2 0PT, UK
[9]British Heart Foundation Centre of Excellence, Cambridge Biomedical Campus, Long Road, Cambridge CB2 0QQ, UK
[10]Département d'Informatique, Université de Sherbrooke, Sherbrooke, QC J1K 2R1, Canada
[11]Département de Biologie, Université de Sherbrooke, Sherbrooke, QC J1K 2R1, Canada
[12]Centre de recherche du Centre hospitalier universitaire de Sherbrooke, Sherbrooke, QC J1H 5N4, Canada
[13]Department of Human Genetics, McGill University, Montréal, QC H3G 1Y6, Canada
[14]Génome Québec Innovation Center, Montréal, QC H3A 0G1, Canada
[15]Institute of Human Genetics, Christian Albrechts University, 24105 Kiel, Germany
[16]Department of Pediatrics, Christian-Albrechts-University Kiel & University Hospital Schleswig-Holstein, 24105 Kiel, Germany
[17]Institute of Human Genetics, University of Ulm, Albert-Einstein-Allee 11, 89081 Ulm, Germany
[18]Department of Hematology, University of Groningen and University Medical Center Groningen, PO Box 30001, 9700 RB Groningen, the Netherlands
[19]Department of Biochemistry, PMAS Arid Agriculture University Rawalpindi, 46300 Rawalpindi, Pakistan
[20]Department of Molecular Biology, Faculty of Science, Nijmegen Centre for Molecular Life Sciences, Radboud University, 6500 HB Nijmegen, the Netherlands
[21]Lead Contact
*Correspondence: c.breeze@ucl.ac.uk (C.E.B.), s.beck@ucl.ac.uk (S.B.)
http://dx.doi.org/10.1016/j.celrep.2016.10.059

SUMMARY

Epigenome-wide association studies (EWAS) provide an alternative approach for studying human disease through consideration of non-genetic variants such as altered DNA methylation. To advance the complex interpretation of EWAS, we developed eFORGE (http://eforge.cs.ucl.ac.uk/), a new standalone and web-based tool for the analysis and interpretation of EWAS data. eFORGE determines the cell type-specific regulatory component of a set of EWAS-identified differentially methylated positions. This is achieved by detecting enrichment of overlap with DNase I hypersensitive sites across 454 samples (tissues, primary cell types, and cell lines) from the ENCODE, Roadmap Epigenomics, and BLUEPRINT projects. Application of eFORGE to 20 publicly available EWAS datasets identified disease-relevant cell types for several common diseases, a stem cell-like signature in cancer, and demonstrated the ability to detect cell-composition effects for EWAS performed on heterogeneous tissues. Our approach bridges the gap between large-scale epigenomics data and EWAS-derived target selection to yield insight into disease etiology.

INTRODUCTION

Common complex diseases, such as autoimmune, metabolic, and neurodegenerative diseases as well as cancer, typically involve multiple genetic and non-genetic factors. The search for genetic factors through genome-wide association studies (GWAS) has identified thousands of replicated SNPs associated with many diseases and other complex phenotypes (Welter et al., 2014). Notably, most of the identified variants were found to map at non-protein coding regions where their molecular consequences are difficult to evaluate (Paul et al., 2014). The search for non-genetic factors is more complicated due to the many

This is an open access article under the CC BY license (http://creativecommons.org/licenses/by/4.0/).

confounding factors, in particular, genetic heterogeneity between individuals and cell-type heterogeneity in accessible sample material (e.g., whole blood). Nonetheless, significant progress has been made following the introduction of systematic epigenome-wide association studies (EWAS) in 2011 (Rakyan et al., 2011), including correction of cell-composition effects (Houseman et al., 2012; Zou et al., 2014; McGregor et al., 2016; Houseman et al., 2016). Since then, over 250 EWAS have been conducted (Michels et al., 2013). A large proportion of these EWAS have correlated DNA methylation (DNAm) profiles with disease status and environmental exposure in cross-sectional case-control cohorts. A subset of these studies reported differentially methylated positions (DMPs) that replicated in independent sample cohorts, e.g., for smoking behavior (Philibert et al., 2015). Fine mapping of the disease-associated genetic variants and integrative analysis with cell type-specific epigenomic datasets suggest a possible causal involvement of affected regulatory sequences, especially enhancers (Farh et al., 2015; Kundaje et al., 2015).

Several large-scale initiatives such as the ENCODE (ENCODE Project Consortium, 2012), Epigenomics Roadmap (Kundaje et al., 2015), and BLUEPRINT (Adams et al., 2012) projects have mapped gene regulatory elements across a wide range of tissues and cell types using a variety of assays. Active regulatory elements have been shown to concentrate at DNase I hypersensitive sites (DHSs) (Dorschner et al., 2004). The intersection of these DHSs with common disease-associated variants has proved to be a powerful approach to identify potential regulatory variants implicated in the disease (Maurano et al., 2012). This approach has been implemented as a publicly available web tool, termed FORGE, and systematically applied to analyze SNP sets for 260 phenotypes from the GWAS catalog (Dunham et al., 2015). The functional interpretation of the resulting SNP-DHS overlaps is that highly associated GWAS SNPs are enriched for DHSs of specific cell types that are relevant to the etiology of the disease under investigation. This is because such SNPs are thought to exert functional changes in affected regulatory elements (marked by DHSs), which, in turn, can then affect gene expression and may ultimately result in phenotypic changes.

While genotypes are generally invariable, epigenotypes are ontogenetically, spatially, and temporally variable. This can impede the meaningful interpretation of DMPs identified through EWAS (Michels et al., 2013; Birney et al., 2016; Paul and Beck, 2014). For example, EWAS are often performed on heterogeneous tissues (e.g., whole blood) where cell-composition effects, or only a specific cell type within that tissue, may be driving the observed epigenetic signal. While significant progress has been made to reduce major sources of confounding (reviewed in Liang and Cookson, 2014; Wilhelm-Benartzi et al., 2013; Rakyan et al., 2011; Michels et al., 2013; Birney et al., 2016), additional tools are required to improve EWAS analysis and elucidate disease mechanisms. To this end, we have developed eFORGE (experimentally derived Functional element Overlap analysis of ReGions from EWAS), a bioinformatics tool that informs which EWAS DMPs are likely to be functional and in which tissue or cell type. This is achieved by overlap analysis between a DMP set of choice and reference DHS maps, improving on previous overlap analyses (Stadler et al., 2011; Slieker et al., 2013). eFORGE has been implemented both as standalone software and an interactive web tool, and rigorously tested for performance criteria, including reproducibility, false-positive rates, and code execution speed. To demonstrate its utility, we systematically analyzed publicly available EWAS datasets to explore the suitability of eFORGE for analyzing surrogate tissues and detecting cell-composition effects, with the ultimate aim of providing insights into disease mechanisms.

RESULTS

eFORGE Method and Workflow

The main objective of eFORGE is the prediction of disease-relevant cell type(s) from EWAS data generated in heterogeneous tissues, as illustrated in Figure 1A. eFORGE analyses such EWAS data generated using Illumina Infinium BeadChips (i.e., 27k, 450k, and EPIC arrays). For a given set of high-scoring probes on the array platform (indicated by large black dots in upper left panel), eFORGE generates a background of 1,000 random probe sets with matching properties regarding their location within genes and CpG islands (Tables S1 and S2). It then overlaps these sets with DHSs for up to 454 selectable reference samples (tissues, primary cell types, and cell lines) from the ENCODE, Epigenomics Roadmap, and BLUEPRINT projects (Data S1). Finally, eFORGE compares the number of DMPs overlapping DHSs with those obtained by random probes to calculate enrichment scores for each of the selected cell types. For a given eFORGE plot, as illustrated in the bottom right panel of Figure 1A, cell types are shown on the horizontal axis and the significance (e.g., –log10 binomial p value) is shown on the vertical axis. Each dot represents an enrichment p value for a given cell type. Enrichments with multiple testing-corrected q values <0.05 and <0.01 are shown as pink and red dots, respectively. Samples that do not reach statistical significance (q value >0.05) are shown in blue.

Assessment of eFORGE Performance

First, we assessed the reproducibility of eFORGE. Each time eFORGE is run on a dataset, it selects matched background probes at random. Because of this, the background is different for each eFORGE analysis. To assess the level of reproducibility between individual analyses, we compared 1,000 independent eFORGE runs using the same dataset, which also represents the default test set made available as part of the stand-alone and web-based versions of eFORGE (see also Experimental Procedures). Using this test set, we obtained a highly consistent pattern of enrichment for the same target cell types between individual runs (Figure 1B), demonstrating that eFORGE results are highly reproducible.

Second, we assessed two methods for the correction of multiple testing: Benjamini-Hochberg (BH), a false discovery rate (FDR)-based correction method for independent tests (Benjamini and Hochberg, 1995), and Benjamini-Yekutieli (BY), an FDR-based correction for multiple tests that are not independent (Benjamini and Yekutieli, 2001). We reasoned that the latter would be more appropriate in our case as DHSs across different cell types have been found to be cell lineage dependent (Stergachis et al., 2013). Benjamini-Hochberg correction resulted in one

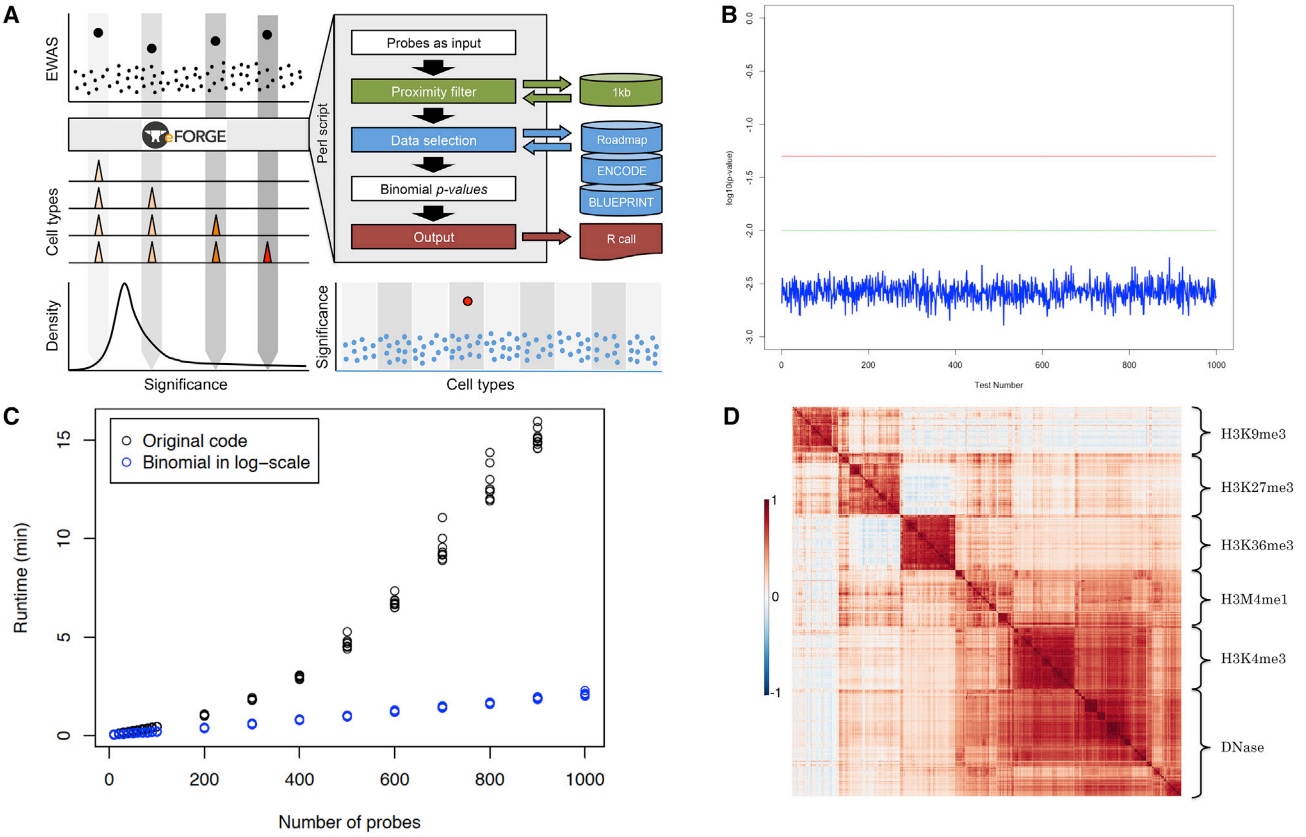

Figure 1. eFORGE Overview and Performance

(A) Concept, components, and flowchart of eFORGE: upper-left panel depicts typical EWAS results with top hits marked as large black dots that serve as input for eFORGE. The main components of eFORGE are controlled by Perl software that uses data from the Roadmap Epigenomics, ENCODE, and BLUEPRINT projects to compute enrichment and significance profiles (illustrated by middle and bottom left panels). R code is used to generate output graphs (illustrated by bottom right panel) with predicted target cell types marked in red.

(B) Reproducibility: using the $CD14^{+}$ tDMP dataset (Jaffe and Irizarry, 2014), 1,000 different runs were performed showing that the variability due to random background sampling is well below the two eFORGE thresholds (green and red lines) that affect target prediction (shown in log scale). These data indicate high reproducibility between eFORGE runs.

(C) Runtime: comparison of Perl BigFloat and BigInt (original code, in black) versus logarithm-based code (in blue) for the management of decimal p value numbers shows up to a 15-fold increase in speed for logarithm-based code. Original code was unable to process 1,000 probes, so data are only shown for probe sets under 1,000 probes.

(D) GeEC correlation data matrix for DHS/Histone mark data from the Roadmap, ENCODE, and BLUEPRINT projects. Red regions show high positive correlation (as measured by Pearson correlation coefficient), white regions show no correlation and blue regions show high negative correlation. Grouping of data by hierarchical clustering agrees with original DHS/Histone mark label (y axis), suggesting a similarity in measurements between different consortia. See also Tables S1–S5, S6 and Data S1.

false-positive (q value <0.01), after running >2.3 million tests (8,000 runs of 299 samples each), compared to no false-positives with BY correction (q value <0.01). Based on this result, we implemented BY correction for eFORGE analyses (see also Tables S3 and S4).

Third, we assessed eFORGE computational speed using different approaches to manage the large decimal p value numbers resulting from the many statistical tests performed. The Perl BigFloat and BigInt functions used by FORGE (Dunham et al., 2015) were slow and, in some cases, even impeded eFORGE code execution. Switching to using the logarithms of the values for the binomial test not only reduced the amount of digits needed in calculation, but also dramatically increased code execution speed as shown in Figure 1C. We anticipate that this advance will become particularly noticeable in the online version of eFORGE when encountering high user demand.

Inter-consortium Correlation Analysis

One possible caveat when interpreting eFORGE results could be consortium-specific differences in the generation of reference datasets used by eFORGE. These include data-analysis pipelines, experimental protocols (including read depth specifications), and materials used (i.e., ENCODE predominantly analyzed transformed cell lines, whereas Roadmap and BLUEPRINT used primary tissue samples). In order to quantify any consortium bias on data generation, we applied the Genomic Efficient Correlator (GeEC) tool (see Experimental Procedures). This tool can measure the correlation of data from different consortia and identify drivers of clustering for

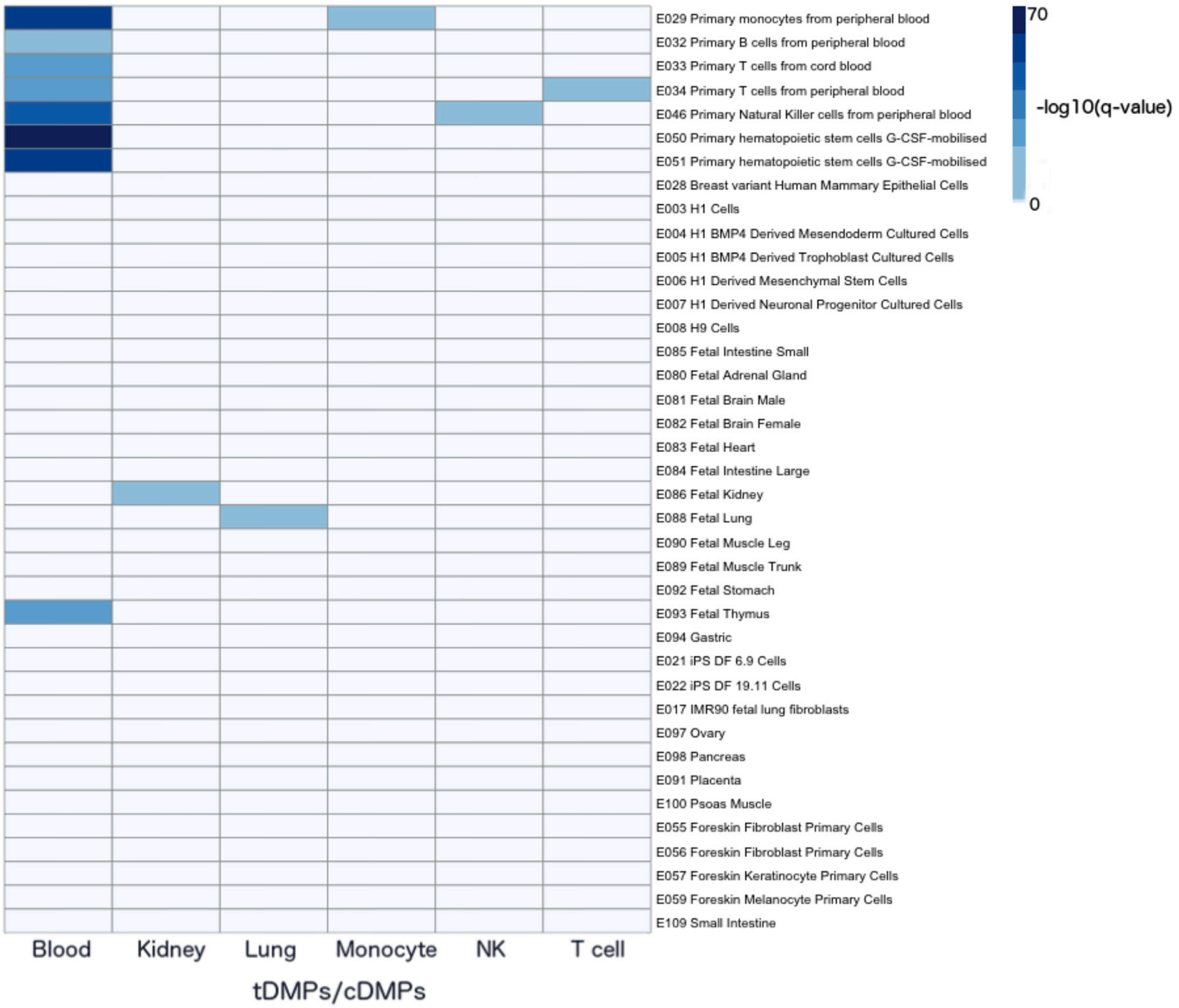

Figure 2. eFORGE Analysis of tDMPs and cDMPs

Results show ability to predict target tissues from known tissue-specific differentially methylated positions (tDMPs) and cell type-specific DMPs (cDMPs): the heatmap is a composite of results for the top 1,000 tDMPs for blood, kidney, and lung (Lowe et al., 2015), and top cDMPs for $CD14^{+}$, T cells, and NK cells (Jaffe and Irizarry, 2014). With tDMPs and cDMPs, we have the advantage of a known prior tissue- or cell type-specific association. We can thus test whether the eFORGE tool identifies the correct tissue. The color-coded enrichment results show that eFORGE identified the correct tissue or cell type each time, with no false-positives. This confirms the tool can signal when regions are associated by DNAm with a specific cell type. See also Figure S1 and Data S2, S3, S4, and S5.

these data. This analysis did not detect any consortium bias on data generation (Figure 1D; Table S5).

Application to tDMPs

As a positive control, we assessed the ability of eFORGE to identify the correct tissues and cell type(s) when tested with probe sets of established tissue and cell-type specificity. In this regard, we analyzed three sets of previously reported tissue-specific DMPs (tDMPs) (Lowe et al., 2015) and three sets of cell type-specific DMPs (cDMPs) (Jaffe and Irizarry, 2014) using consolidated Roadmap DHS data. Figure 2 shows the resulting heatmap, which demonstrates the ability of eFORGE to unambiguously identify the relevant tissues and cell types for each tDMP and cDMP set (i.e., blood, kidney, lung, monocytes, natural killer cells, and T cells). To quantify the level at which eFORGE can detect mixed tissue- and cell type-specific enrichment, we next assessed its performance on mixed tDMP and cDMP probe lists. Figure S1A shows the result for a mixture of tDMPs from lung and kidney tissues (Lowe et al., 2015). Although both tissues were predicted correctly in eFORGE, the tissue-specific signal was lower, due to a lack of specific enrichment for the mixed sets in each of the cell types. Figure S1B shows the corresponding results for mixed cDMPs. Here, sets of 148 B cell-specific and 148 monocyte-specific cDMPs (Jaffe and Irizarry, 2014) were mixed, and again the corresponding cell types were

correctly predicted. Taken together, we provided evidence that eFORGE can identify the correct target tissues and cell types from individual and mixed probe sets.

Application to EWAS Data

Next, we applied eFORGE to analyze published EWAS data. First, we considered all EWAS compiled in a review article (Michels et al., 2013) that analyzed at least 100 samples using Illumina Infinium BeadChips. This qualified 44 datasets for eFORGE analysis, of which 20 showed eFORGE signal (q value <0.05). 14 studies showed an enrichment pattern specific to particular tissues. For instance, we observed blood-specific patterns for six blood-based EWAS, and stem cell-specific patterns for five cancer and aging EWAS (Figure 3). The genome-wide distribution of hits from these studies is shown in Figure 4. In addition, we found a larger average sample size for studies that present eFORGE signal (average n = 527) compared to those studies that did not (average n = 191). Taken together, these results suggest that tissue-specific enrichment patterns are widespread among EWAS and that eFORGE demonstrates the capacity to detect these patterns.

Second, to provide specific examples of eFORGE analysis, we assessed the ability of eFORGE to predict disease-relevant cell types from EWAS conducted on immune blood cells for three autoimmune diseases: rheumatoid arthritis (RA) (Liu et al., 2013), systemic lupus erythematosus (SLE) (Coit et al., 2013), and Sjögren's syndrome (Altorok et al., 2014). For these diseases, it is assumed that blood is the main affected tissue. We performed eFORGE analyses for each of these diseases using the reported top 100, 86, and 753 probes and consistently found tissue-specific enrichment for immune effector cells and thymus (Figure 5). Specifically, eFORGE results for the EWAS on RA pointed to $CD14^+$ cells as the most highly enriched cell type (q value = 5.53e-04), indicating a tendency for cell-composition-corrected RA-associated DNAm changes to co-locate with $CD14^+$ DHSs. Indeed, the accelerated maturation of $CD14^+$ cells is a hallmark in RA (Hirohata et al., 1996). For the EWAS on SLE, we observed confirmatory enrichment in DHSs specific to T cells (q value = 2.56e-05). T cells, in particular $CD4^+$ T cells, play an essential role in the development of SLE (Yin et al., 2015) and have previously been proposed as targets for the treatment of Sjögren's syndrome (Singh and Cohen, 2012). Consistent with these findings, eFORGE also pointed to an independent T cell signal for DMPs identified in the Sjögren's syndrome EWAS (q value = 1.31e-49).

Third, we assessed whether eFORGE can uncover patterns in published EWAS data that would inform the functional interpretation of the statistical findings. Using the top 1,000 hypomethylated regions for an EWAS on multiple sclerosis (MS) (Huynh et al., 2014), we generated eFORGE plots for several DHS reference sets (Figure 6A). We observed a tissue-specific enrichment in immune cells, which is unexpected for a study performed on pathology-free brain tissue. To support this observation, we carried out additional eFORGE analysis using histone marks (Kundaje et al., 2015) that showed an enhancer-specific signature (H3K4me1) underlying this DHS enrichment (Figure 6B). We then intersected the top 1,235 hypomethylated regions from the study that gave rise to the observed immune signal with the locations of active enhancers (n = 1,158) previously identified in microglial cells (Lavin et al., 2014). These immune cells constitute up to 15% of all cells in the mammalian CNS (Xavier et al., 2014). A Fisher's exact test confirmed significant co-localization of the microglial-specific active enhancers (p value: 2.70e-07, odds ratio [OR]: 5.88, 95% confidence interval [CI]: 3.19–9.96), suggesting that microglial enhancers may be potential drivers of the MS EWAS signal. In conclusion, eFORGE analysis of published MS EWAS data uncovered tissue-specific patterns, suggesting potential molecular mechanisms relevant to the etiology of the disease.

Fourth, we examined whether eFORGE can be used for the interpretation of EWAS that use surrogate tissues. In these studies, DNAm changes are measured in easily accessible tissues such as whole blood or buccal cells rather than the target tissue that is most relevant to the disease. It has been suggested that DNAm changes in surrogate tissues reflect epigenomic perturbations found in the target tissue (Lowe and Rakyan, 2014). An alternative possibility is that the observed epigenetic signature does not mimic methylation changes in the target tissue but is specific to the surrogate tissue. We performed eFORGE analysis on the top 110 regions from an EWAS on ovarian cancer, which was performed on whole blood using a pre-treatment discovery cohort. We found enrichment for $CD14^+$ cells (q value = 1.37e-12, Figure 6C), but not for ovary (q value = 1) or solid cancer tissues (q value = 1). In addition, the observed myeloid/lymphoid enrichment patterns suggested cell-composition effects, as only myeloid regions showed enrichment, raising the possibility that these DNAm differences were caused by an increase in myeloid cell numbers in one of the groups. While a similar immune signature had been identified before, in addition to cell-composition effects (Teschendorff et al., 2009; Houseman et al., 2012; Li et al., 2014), we used an alternative approach to support this finding. Specifically, we were able to exclude ovarian signal and provide evidence for which myeloid cell types may drive the EWAS signal. For example, DHS enrichment was not observed for megakaryocytes (Figure 6C). In summary, eFORGE analysis of the top hits from an EWAS on ovarian cancer pointed to cell-composition effects driven by a myeloid-specific immune response to this cancer type, rather than an epigenetic change in peripheral blood that mimics a change in the methylome of the ovarian cancer tissue.

Finally, we showed that eFORGE detects tissue-specific patterns in cancer EWAS data. We analyzed five cancer EWAS: breast cancer (Fang et al., 2011), colorectal cancer (Kibriya et al., 2011), sporadic colorectal cancer (Laczmanska et al., 2013), clear cell renal cell carcinoma (Arai et al., 2012), and adrenocortical carcinoma (Barreau et al., 2013). Using the top 330, 450, 240, 801, and 362 EWAS hits, respectively, we performed eFORGE analysis for each DMP set, and identified enrichment in stem cells but not in breast, intestine, or renal tissues across the five studies (Figure 7). This suggests that many regulatory elements affected by cancer epigenetic reprogramming may be stem cell like. This is consistent with previous findings that DNAm changes in cancer tissue aid the emergence of a possible stem cell phenotype (Widschwendter et al., 2007). In conclusion, application of eFORGE to cancer EWAS data provided evidence

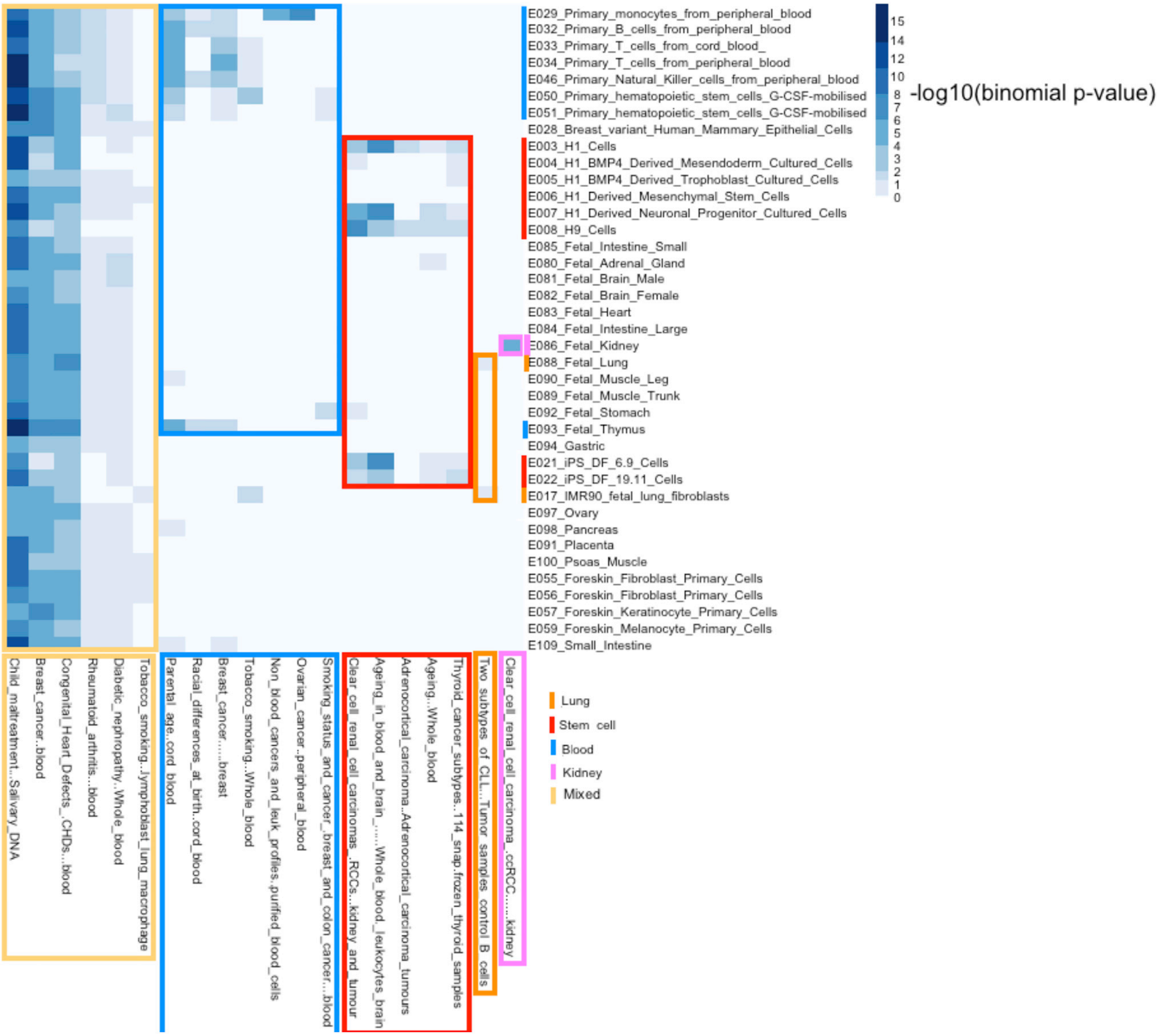

Figure 3. Aggregated Enrichment Statistics for Studies with eFORGE Signal from a Recent Review

Studies were obtained from the review by Michels et al. (2013). This heatmap shows the enrichment statistics (presented as –log10(binomial p value)) for an unbiased selection of EWAS (n = 20 studies, each with at least 100 samples). Many of these studies show an enrichment pattern specific to particular tissues, such as blood (blue box, seven studies) and stem cells (red box, five studies). In addition, one ccRCC study shows a kidney specific enrichment and one CLL study presents a lung-specific enrichment (lung tissue and IMR90). Other patterns are more mixed (yellow box, six studies). Of the seven blood-enriched studies, six were performed in blood and one was performed in breast cancer tissue, which may contain immune cells. All five studies that show a stem cell-specific enrichment are exclusively cancer or aging EWAS. Of the six studies that show a mixed enrichment, there is evidence of different components underlying variation. For example, the EWAS on child maltreatment performed on salivary DNA, despite showing enrichment for many tissues, has blood cell types as the highest categories. Work remains to be done to refine these mixed signals and define the components that are driving enrichment for several different tissue types. See also Table S7.

for a stem cell-like enrichment across all five studies, which warrants further investigation.

DISCUSSION

We have developed eFORGE, a tool that highlights DMPs identified through EWAS that are likely to be functional in a cell type- and tissue-specific context. Its development represents an addition to the currently limited toolbox available for comprehensive analysis and interpretation of EWAS data. Both the standalone and web-based versions of eFORGE have been subjected to rigorous performance assessment with regards to false-positive rates, reproducibility, and speed to ensure ability to cope with high user demand.

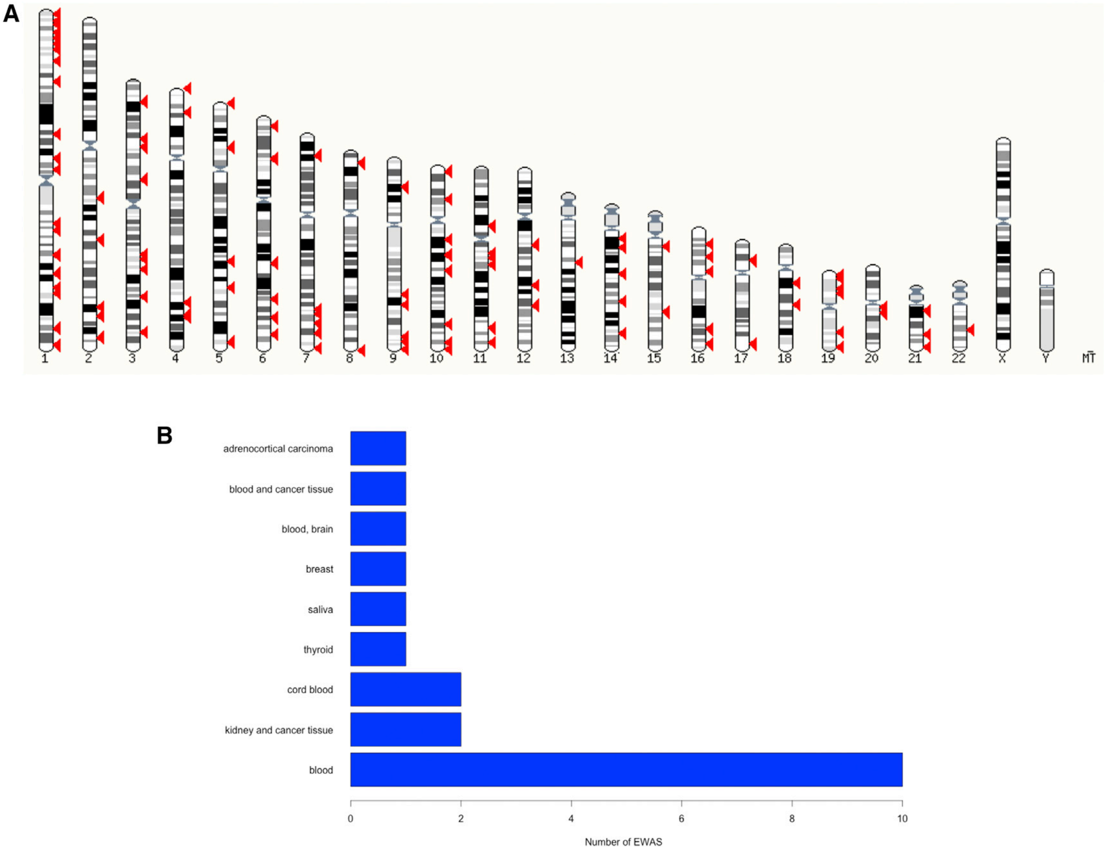

Figure 4. Karyotype View of EWAS Hits and Bar Chart of EWAS Tissues

(A) This karyotype view was obtained taking top ten study hits from each of the 20 EWAS with eFORGE signal (taken from Michels et al., 2013) and performed using ensembl KaryoView (http://www.ensembl.org/Homo_sapiens/Location/Genome). Many EWAS exclude probes from sex chromosomes as part of study analysis, and therefore there is an absence of top hits in these chromosomes on the graph. Apart from this, there seems to be no strong bias in the distribution of EWAS hits along the genome.

(B) Bar chart indicating analyzed tissue for 20 EWAS with eFORGE signal from Michels et al. (2013). As is to be expected for an easily accessible tissue, blood is the most analyzed category, with ten studies.

See also Table S7.

SNPs identified through GWAS have been systematically probed for enrichment at regulatory elements marked by DHSs (Maurano et al., 2012). This analytical approach has recently been implemented in the FORGE tool, which enables an automated analysis workflow (Dunham et al., 2015). Complementing FORGE for epigenetic analysis, we have developed eFORGE to provide large-scale, tissue-specific DHS enrichment analysis for DMPs identified through EWAS. While using parts of the FORGE framework (Dunham et al., 2015), eFORGE uses a different, EWAS-specific background and contains several features not included in FORGE, such as a faster scaling of the binomial test and histone marks as a dataset additional to DHSs.

The main applications of eFORGE are the analysis of EWAS data to predict disease-relevant cell types and potential cell-composition effects, as well as quality-control analysis for studies on tissue-specific DNA methylation. We have provided evidence that eFORGE analysis of tDMPs correctly predicts the relevant tissue through tissue-specific DHS enrichment patterns (Figure 2). In this way, sets of tDMPs can be linked to the corresponding tissue in an independent manner. This link can also be used in the inverse sense to detect regions with a potential tissue-specific regulatory function, by using algorithms to detect probe sets with a high tissue-specific eFORGE score. eFORGE is also designed to aid downstream functional follow-up of DMPs by providing tissue-specific DHS overlap results in the form of tables, with data for specific genomic regions. DHSs are markers of *cis*-regulatory elements (Dorschner et al., 2004), and DNAm changes in these regions may be associated with functional consequences (Schübeler, 2015). Such functional links could be confirmed through experimental assays, including chromatin conformation capture techniques and epigenome editing using CRISPR/Cas9 (Köeferle et al., 2015).

eFORGE can also be used to assess cell-composition effects in EWAS, providing a complementary approach to methylation-based tools. When heterogeneous tissues are analyzed in EWAS, a proportion of the observed differential DNAm signal can be due to cell-composition effects (Houseman et al., 2012, 2016; Paul and Beck, 2014). eFORGE can identify these

OPEN ACCESS
CellPress

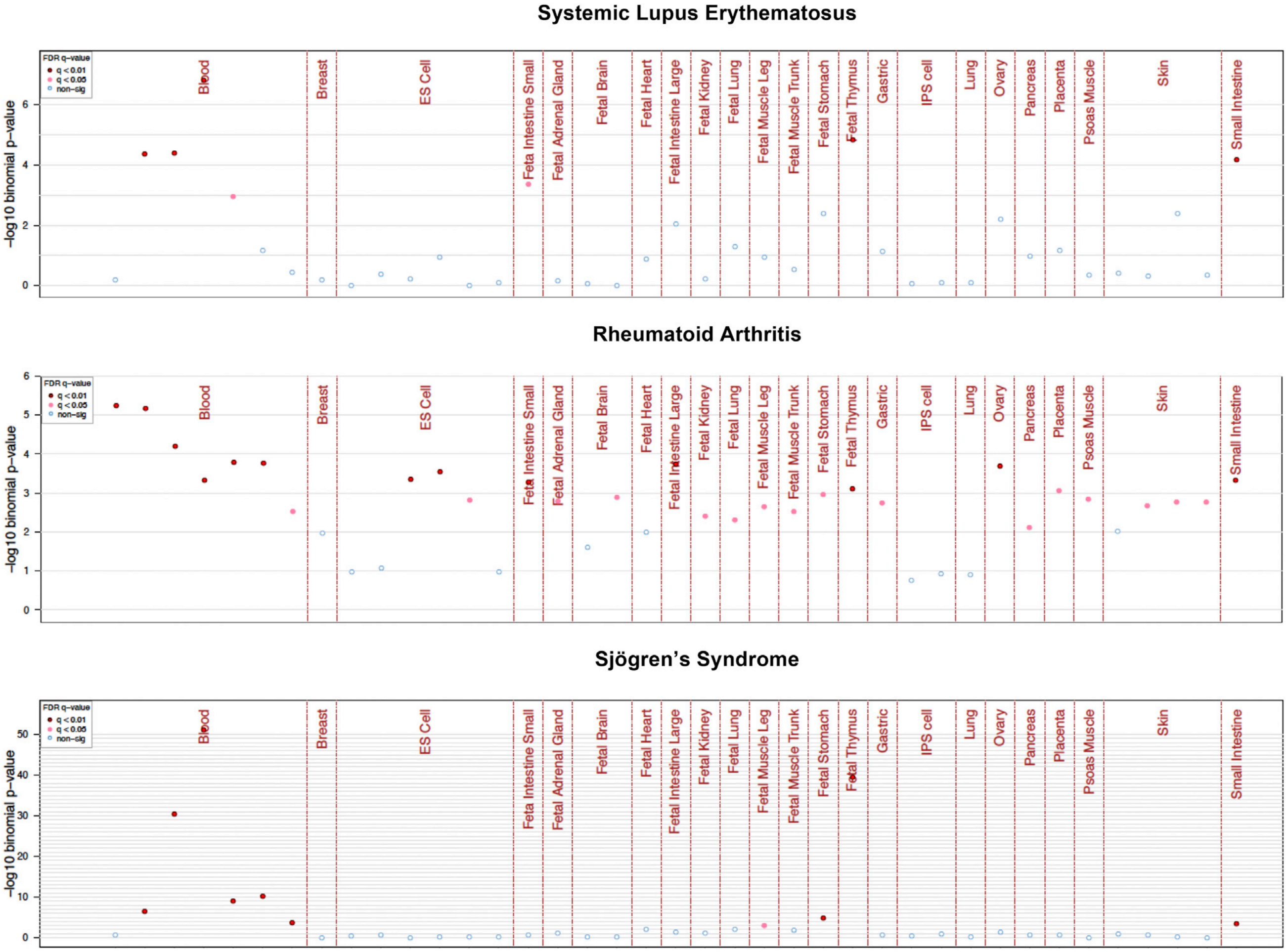

Figure 5. eFORGE Analysis of Autoimmune EWAS

Top panel shows a blood (predominantly T cell), intestine, and thymus-specific signal for 86 probes from an EWAS on SLE. Middle panel shows a more general pattern of enrichment, with a strong blood signal, with $CD14^+$ cells as the highest category, for a set of 100 RA EWAS probes. Bottom panel shows a blood (predominantly T cell) and thymus-specific enrichment for a set of 753 probes for an EWAS on Sjögren's syndrome. Probe lists were obtained from the supplementary files of the studies (Coit et al., 2013; Liu et al., 2013; Altorok et al., 2014).

cell-composition effects by detecting tissue-specific DHS enrichment based on genomic location, not DNAm values. Unlike alternative methods for detecting cell-composition effects (Zou et al., 2014; Houseman et al., 2012, 2016), eFORGE provides the user-friendly option of web-based analysis.

eFORGE analysis is, however, not without limitations. Despite the fact that eFORGE can detect cell-composition effects, the user must interpret whether a given enrichment is driven by confounding cell-composition effects or by true cell type-specific effects. Based on the data presented here, strong bias toward one cell type for an EWAS performed on heterogeneous tissue is more likely an indication of cell-composition than cell type-specific effects. The need for a more accurate interpretation requires complementary methods for cell-composition detection and correction to be used and also highlights the requirement of experimental validation. In addition, eFORGE can only analyze cell types for which DHS data are available. Although the current eFORGE database contains 454 samples, many cell types are still not represented. To consider these, we have to recur to alternative analyses, as shown in our analysis of microglial enhancer enrichment outside of eFORGE.

In conclusion, we anticipate eFORGE to contribute to the challenging task ahead of translating the increasing number of DMPs identified through EWAS into relevant molecular mechanisms.

EXPERIMENTAL PROCEDURES

DNase I Data

DNase I data from the ENCODE, Roadmap Epigenomics, and BLUEPRINT projects were downloaded and, if necessary, processed using the Hotspot method (see Supplemental Experimental Procedures). Fastq and BAM files for BLUEPRINT samples (listed in Table S6) are available at the European Genome-phenome Archive under accession number EGAD00001002713.

EWAS DMP Data

A list of 44 450k- and 27k-based EWAS was analyzed with eFORGE. The list of 20 studies with eFORGE signal is contained in Table S7. Studies were selected

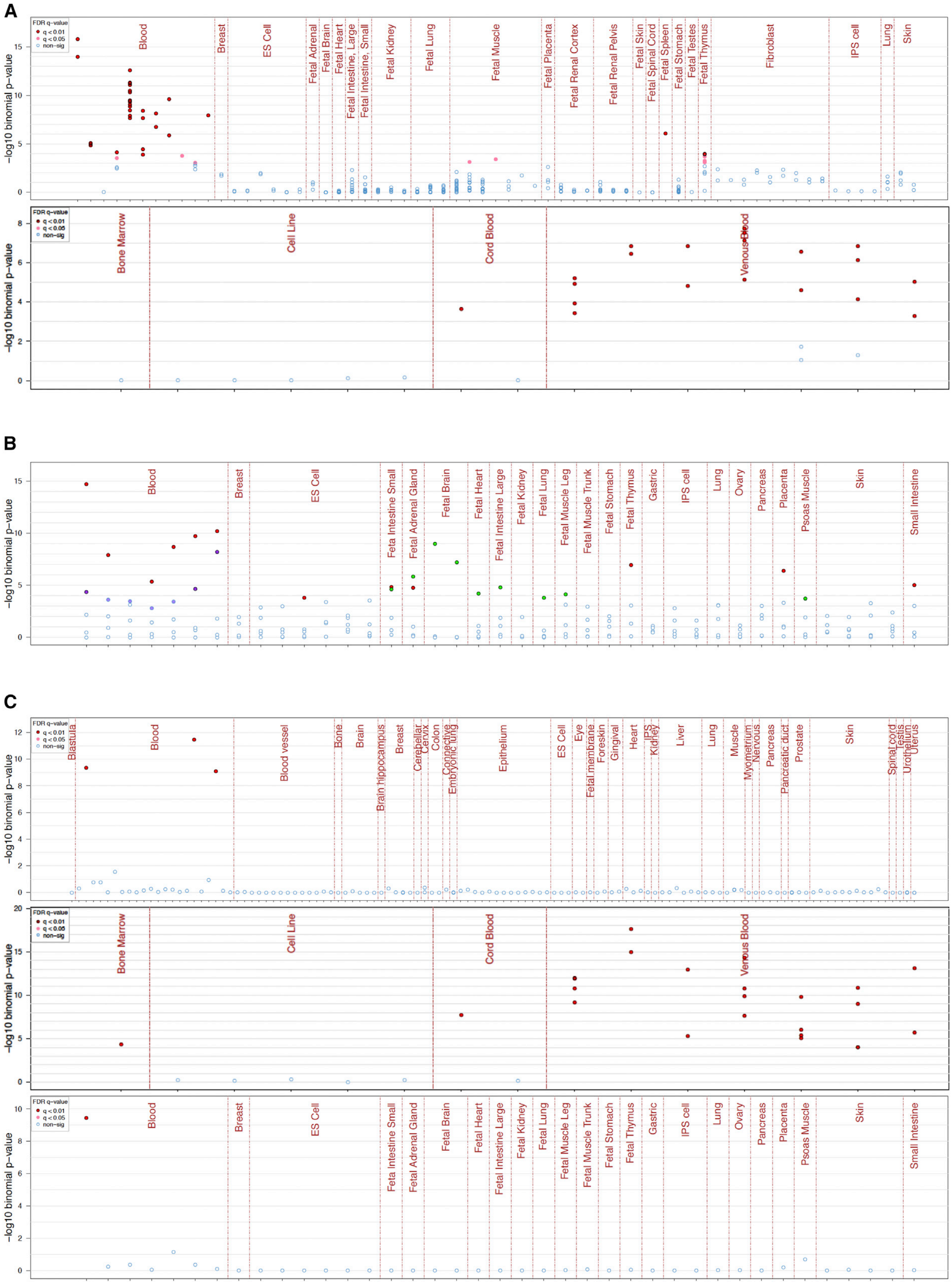

(legend on next page)

from Table S1 of a review on EWAS (Michels et al., 2013), taking only studies with a number of samples equal to or above 100. eFORGE analysis of DMP sets was performed for DNase I hotspots from ENCODE, BLUEPRINT, and Roadmap Epigenome projects, with background DMP sets from the Illumina 450k or 27k array where appropriate. For the 27k array background, only probes shared with the 450k array were used. Notably, 13 of the EWAS selected from the aforementioned review did not report top probes. This lack of reporting top probe IDs is an important and not previously reported finding, constituting a major limitation for EWAS reproducibility, in addition to hindering eFORGE analysis of published studies. We urge the community to embrace the open and clear reporting of EWAS results, including top study hits.

Preparation of eFORGE Overlaps

An SQLite database (http://www.sqlite.org) containing the overlaps for the Infinium Illumina 450k array (Bibikova et al., 2011) cg probes with the BLUEPRINT, ENCODE, and Roadmap Epigenome DNase I hotspots was incorporated into the eFORGE tool. The HumanMethylation450 v.1.2 Manifest File with data from all the cg probes on the 450k array was used to prepare this database (https://support.illumina.com/array/array_kits/infinium_humanmethylation450_beadchip_kit/downloads.html).

We compared the 450k array cg probe data to the indexed DNase I hotspots using the *bedtools* tool (Quinlan and Hall, 2010). The overlaps for each cg probe were stored in an SQLite DB, organized by datasets. For each cg probe, a binary string was generated, representing the presence or absence of an overlap with a hotspot in each dataset (either Roadmap, BLUEPRINT, or ENCODE).

Background Probe Parameters

eFORGE evaluated the input DMP set by comparing it to 1,000 background DMP sets. The probes in the background sets were matched to the probes in the input set using annotation (i.e., if one probe from the input set was in a promoter, then its matching probe in each of the 1,000 background sets was also in a promoter). The annotation categories used for this matching process were "Gene annotation" (i.e., 1stExon, 3′ UTR, 5′ UTR, Body, IGR, TSS1500, TSS200) and CpG Island annotation (i.e., Island, Shore_Shelf, and NA or "open sea"). All possible combinations of these two levels resulted in 21 different background bin categories. The CpG island annotation categories "Shelf" and "Shore" were merged to produce enough probes in each background bin category; i.e., more than 1,000. The 450k array Illumina annotation (Bibikova et al., 2011) was used to create these data categories (Tables S1 and S2).

eFORGE Analysis

Input of DMPs into eFORGE can be in any of two forms: as Illumina 450k/27k probe IDs or as BED format (BED format should be zero based and the chromosome should be given as chrN, as genomic location on human genome assembly GRCh37). Genome coordinates are sufficient to identify probe IDs if these are not provided in BED format. We suggest a minimum of 20 and a maximum of 1,000 probes. If a DMP is not present on the 450k array (or the 27k array probes shared with the 450k array), it is excluded from the analysis. We added a 1-kb proximity filter in order to avoid the biases of testing groups of proximal probes in eFORGE: methylation correlation among closely located CpGs could mean we would be testing the same change more than once. Probes from input are selected at random by the filter, and any probe within 1 kb of any already selected probe is excluded. The choice of selecting 1 kb as a limit for filtering was based on previous data showing strong correlation of DNA methylation levels between CpGs fewer than 1 kb apart (Eckhardt et al., 2006).

Overlaps are retrieved from the eFORGE database for each analyzable probe in the input set. The tool records a count of total hotspot overlaps for each DNase I sample (cell) for the test probe set. eFORGE selects 1,000 matching background probe sets that contain an equal number of probes to the test probe set, matching for gene annotation and CpG island annotation as described above. Retrieval of overlaps from the database for each of the probes in each of the background probe sets then occurs. The tool records an overlap count for each background set in each DNase I sample. For each test probe set, eFORGE obtains the binomial p value for the test set overlap count. This binomial p value is calculated for the test set overlap count relative to the total number of tested probe sets. The binomial test was chosen over the hypergeometric test due to the important computational speed advantages it offers, which are further highlighted considering the high number of tests performed by eFORGE.

eFORGE Outputs

Tabular and graphic descriptions of the enrichment of overlap for the test DMPs are generated by eFORGE for each DNase I hotspot sample. The tool outputs a tab-separated values (TSVs) file, which includes columns for *Z* score, binomial p value, cell, tissue, datatype, filename of the sample hotspots, DMPs that contribute to the enrichment, the GEO accession for each sample, and the BY adjusted q value. An interactive table containing these data is generated using the Datatables (https://datatables.net/) plug-in for the jQuery JavaScript library accessed through the rCharts package (http://ramnathv.github.io/rCharts/).

In terms of graphical visualization of eFORGE output, FORGE scripts (Dunham et al., 2015) were adapted with minor modifications. Briefly, the –log 10 (binomial p value) is presented by cell sample in each of the graphic outputs. Base R graphics (http://www.r-project.org) are used to generate a pdf graphic. eFORGE generates the interactive JavaScript graphic using the rCharts package (http://ramnathv.github.io/rCharts/) to interface with the dimple d3 libraries (http://dimplejs.org). In the pdf and the interactive graphic cells are grouped alphabetically within each tissue (tissues, in turn, also follow alphabetical order). The coloring of results by BY-corrected q value in each of the graphics is consistent, blue (q value >0.05), pink (q value <0.05), and red (q value <0.01).

Figure 6. eFORGE Analysis of Surrogate Tissue and Multiple Sclerosis EWAS

(A) DHS analysis of multiple sclerosis EWAS. Upper panel shows eFORGE blood, spleen, and thymus enrichment in Roadmap Epigenomics data for top 1,000 hypomethylated DMPs (ranked in the study by likelihood ratio test and Fisher's method FDR q value). Lower panel shows enrichment for macrophages and monocytes in an analysis of the same regions with BLUEPRINT data.

(B) Histone mark analysis of multiple sclerosis EWAS. Panel shows enrichment for top 1,000 study hypomethylated DMRs. Cell type-specific scores are colored by FDR q value. Cell types with q values below 0.01 for histone modifications representative of enhancers (H3K4me1) are shown in red, promoters (H3K4me3) are shown in purple, and polycomb-repressed regions (H3K27me3) are shown in green. Cell types with q values between 0.01 and 0.05 for the histone modification representative of promoters (H3K4me3) are shown in light purple. Cell types with q values above 0.01 for all other histone modifications are shown in blue. H3K36me3 (transcribed regions) and H3K9me3 (a marker for heterochromatin) did not present any significant cell type-specific enrichment patterns. Analyzed regions show enrichment for H3K4me1 (and, at a lower level, H3K4me3) in blood cells.

(C) Analysis of surrogate tissue EWAS: the three panels (ENCODE, BLUEPRINT, and consolidated Roadmap, from top to bottom) show enrichment for monocyte, macrophage, and AML for an ovarian cancer prediction EWAS measured on whole blood. There is no enrichment for any other tissue (including lymphoid cells, ovarian tissue, and, interestingly, megakaryocytes). This supports a myeloid-lineage-specific DHS enrichment for top regions from this EWAS. By discarding enrichment in megakaryocyte regions, and showing enrichment for acute promyelocytic leukemia cell lines (NB4 and HL-60), the lineage-specific component of this tissue-specific signal points to a divergence that occurs after differentiation from the common myeloid progenitor and is suggestive of an event during myeloblastic differentiation. This DHS enrichment pattern extends to the myeloblast branch of the myeloid lineage, pointing to these regions being active in the myeloblast, which would be the cell of origin of this tissue-specific signal. This enrichment pattern shows cell types that drive the proposed myeloid/lymphoid imbalance causing the methylation signal observed (Teschendorff et al., 2009; Houseman et al., 2012; Li et al., 2014).

OPEN
ACCESS
CellPress

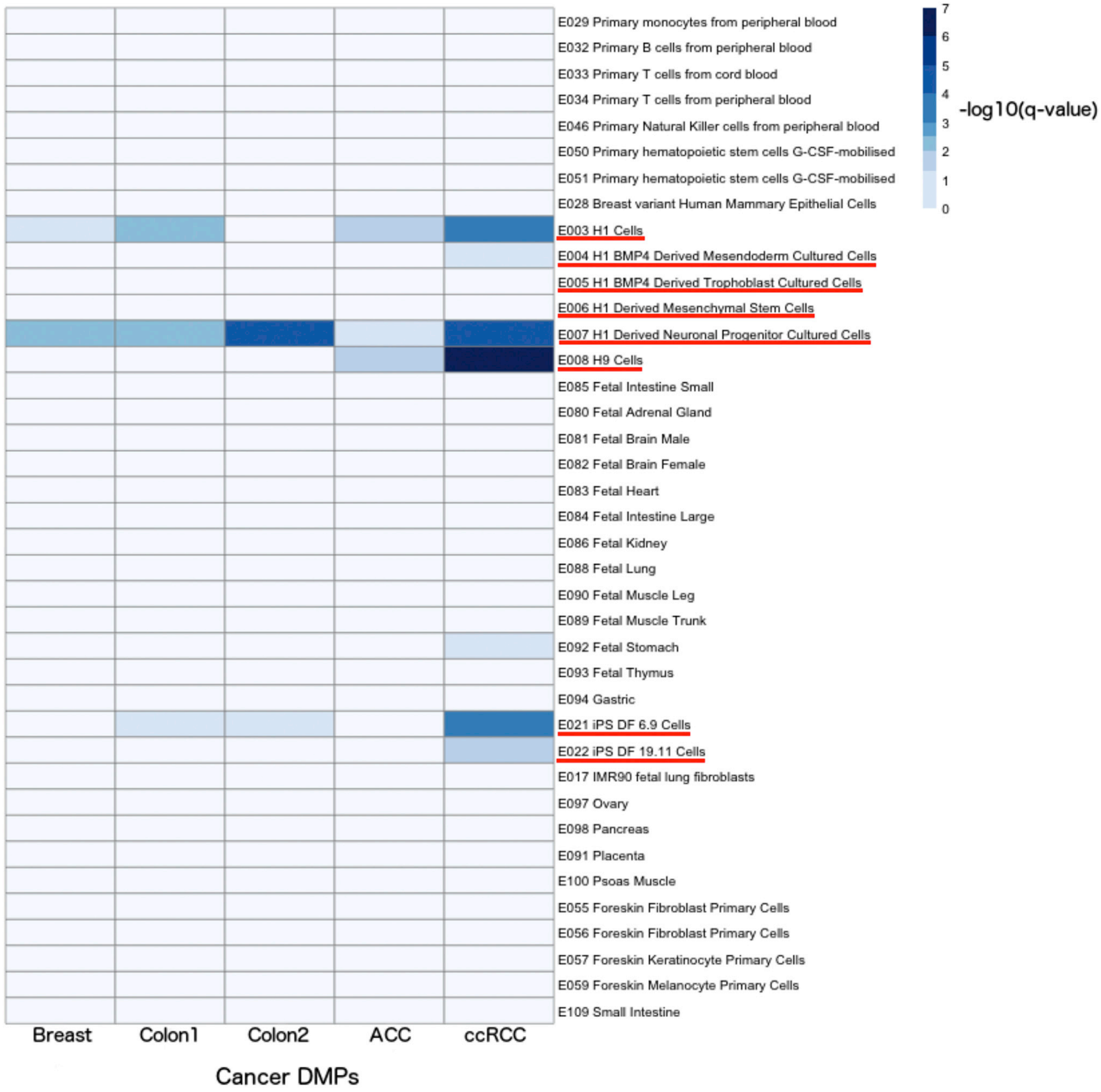

Figure 7. Analysis of Cancer EWAS

This heatmap shows a stem cell-like signature for regions from five cancer EWAS, through color-coded enrichment –log10(q value). The left column depicts results for 330 top probes from a breast cancer metastatic behavior EWAS (Fang et al., 2011), the second column from the left shows results for 450 probes from a colorectal carcinoma EWAS (Kibriya et al., 2011), and the central column shows results for 240 probes from a sporadic colorectal cancer EWAS (Laczmanska et al., 2013). The next column on the right shows results for 801 probes from an adrenocortical carcinoma EWAS (Barreau et al., 2013), and the last column on the right shows results for 362 probes from a clear cell renal cell carcinoma EWAS (Arai et al., 2012). All five studies showed intermediate enrichment (q value <0.05) of at least one eFORGE "ES cell" or "iPSC" category. Aside from this stem cell-like signature, no other shared tissue category is enriched across the five probe lists.

eFORGE Reproducibility

A default probe set of 11 monocyte-specific DMPs was run 1,000 times on eFORGE, and we plotted a resulting longitudinal set of $CD14^+$ category q values for these runs (Figure 1B).

False-Positive Testing

We tested eFORGE BY correction with sets of 5, 10, 15, 20, 30, 40, 50, and 100 randomly selected probes, performing 5,000 analyses for both Roadmap Epigenomics and ENCODE data. We did not obtain any false-positives (at a q

value <0.01) for any of these analyses. We did, however, obtain false-positives at a q value <0.05 (0.36%), but this is only indicated to be an intermediate level of significance in eFORGE. The false-positive rates at a q value <0.05 do not show a clear tendency with the size of the probe sets (see Tables S3 and S4). Most of the q values below 0.05 come from a few probe sets, signaling that most FP come from random sets that are borderline for many tissues/cell lines. This supports the notion that it is highly improbable to obtain eFORGE tissue-specific enrichment with random probe lists.

Comparison of BH and BY

8,000 sets ranging between five and 100 DMPs were run on eFORGE using both BH and BY multiple testing correction methods. As 299 samples were analyzed for each of the 8,000 DMP sets, there is a total number of 2,392,000 sample tests. Out of these random tests, one false-positive was recorded for BH, and zero were recorded for BY, at a significance level of 0.01.

Addition of New Data to eFORGE Database

All the code for generating the eFORGE database from scratch is openly available on GitHub (see https://github.com/charlesbreeze/eFORGE/blob/master/database/README.txt and examples in the same directory). Data from a new project can be added as a table to the modular SQLite database. In addition, indications are provided for the production of bitstring tables from raw data.

GeEC Analysis

Datasets used for GeEC correlation analysis were taken from the IHEC Data Portal (http://epigenomesportal.ca/ihec/) as of March 2016. They correspond to 508 DNase sequencing (DNase-seq) datasets generated by ENCODE, Roadmap, and BLUEPRINT, as well as 1,277 chromatin immunoprecipitation sequencing (ChIP-seq) datasets for five core histone marks (H3K4me1, H3K4me3, H3K36me3, H3K27me3, and H3K9me3) generated by ENCODE and Roadmap. These 1,785 datasets were processed with the Genomic Efficient Correlator (GeEC) tool to first average the signal of each dataset in non-overlapping bins of 10 kb (excluding the ENCODE blacklisted regions) and then to calculate a matrix of the pairwise Pearson product-moment correlation coefficients (r). Hierarchical clustering (using the 1-r distance metric and the average linkage method) was then conducted, and the principal clusters were annotated.

Adjusted Rand Index (ARI) results were also used to validate the accuracy of the clustering by using the IHEC-provided metadata describing every dataset for three categories of labels (assayType, cellType, consortium). The tree generated through hierarchical clustering is cut at the appropriate height to obtain a number of clusters equal to the number of distinct labels in each category and then used to calculate the ARI scores. ARI values were calculated using the "average" linkage method.

tDMP Analysis

We extracted normalized samples from Marmal-aid (Lowe and Rakyan, 2013) for tissues that contained a minimum of 50 samples. We used the dmpFinder function in minfi (Aryee et al., 2014) to make an initial call of tissue-specific methylation differences. Two categories were defined to perform this for each tissue: group 1 contained the tissue of interest, and other tissues were included in group 2. We used the top two probes for each of the tissue-specific calls to visually inspect the data. Any samples that were found to be closer to the mean of group 2 than group 1 were removed. The final calls were then produced using the remaining samples. We randomly selected 50 samples for each tissue, and we then followed a similar procedure to the above, calling differences using dmpFinder. Data S2 contains the IDs of the samples used in the analysis. Data S3, S4, and S5 contain the calls for three of the tissues for all probes.

cDMP Analysis

We obtained data from Table S2 of a cell type-specific methylation paper by Jaffe and Irizarry (2014). cDMP selection was based on a given probe having the lowest methylation value for a given cell type when compared to the other cell types, and this low methylation value being lower than the next closest methylation value by 0.4.

Source Code

The source code for eFORGE is available on GitHub at https://github.com/charlesbreeze/eForge. It includes the code for the standalone tool, the web-server, and the scripts to build or extend the database, although a pre-compiled eForge.db sqlite database and background selection hash tables are readily available at http://eforge.cs.ucl.ac.uk/?download. Code variants (e.g., probe weighting) are discussed in the Supplemental Experimental Procedures. eFORGE has been successfully installed and run on Red Hat Linux and OS X 10.9.5.

ACCESSION NUMBERS

The accession number for the BLUEPRINT data reported in this paper is EGA: EGAD00001002713.

SUPPLEMENTAL INFORMATION

Supplemental Information includes Supplemental Experimental Procedures, seven figures, seven tables, and five data files and can be found with this article online at http://dx.doi.org/10.1016/j.celrep.2016.10.059.

AUTHOR CONTRIBUTIONS

C.E.B., D.S.P., I.D., E.B., J.H., and S.B. designed the project. J.v.D., L.M.B., R.L., V.K.R., M.F., K.D., W.H.O., E.V., S.S., F.M., A.K.B., R.S., J.L., P.-E.J., GB, J.H.A.M., and H.G.S. provided data. C.E.B., I.D., and J.H. designed and developed the software package with support from A.E.T., J.C.A., J.E.B., and V.I. C.E.B., S.B., and D.S.P. wrote the paper with contributions from all authors.

ACKNOWLEDGMENTS

C.E.B. was supported by a PhD fellowship from the EU-FP7 project EpiTrain (316758). J.H. was supported by the UCL Cancer Institute Research Trust. V.K.R. was supported by BLUEPRINT (282510). K.D. was funded as a HSST trainee by NHS Health Education England. M.F. was supported by the BHF Cambridge Centre of Excellence (RE/13/6/30180). Research in W.H.O.'s laboratory was supported by EU-FP7 project BLUEPRINT (282510) and by program grants from the National Institute for Health Research (NIHR, http://www.nihr.ac.uk) and the British Heart Foundation under numbers RP-PG-0310-1002 and RG/09/12/28096 (https://www.bhf.org.uk/). W.H.O.'s laboratory receives funding from NHS Blood and Transplant for facilities. We gratefully acknowledge the participation of all NIHR Cambridge BioResource volunteers. We thank the Cambridge BioResource staff for their help with volunteer recruitment. We thank members of the Cambridge BioResource SAB and Management Committee for their support of our study and the National Institute for Health Research Cambridge Biomedical Research Centre for funding. R.S. and his group were supported by the European Union in the framework of the BLUEPRINT Project (HEALTH-F5-2011-282510) and the German Ministry of Science and Education (BMBF) in the framework of the MMML-MYC-SYS project (036166B). We thank Deborah Winter (Weizmann Institute) for supplying a set of microglial enhancers from Lavin et al. (2014). Research in S.B.'s laboratory was supported by the Wellcome Trust (99148), Royal Society Wolfson Research Merit Award (WM100023), and EU-FP7 projects EpiTrain (316758), EPIGENESYS (257082), and BLUEPRINT (282510).

Received: February 18, 2016
Revised: August 25, 2016
Accepted: September 30, 2016
Published: November 15, 2016

REFERENCES

Adams, D., Altucci, L., Antonarakis, S.E., Ballesteros, J., Beck, S., Bird, A., Bock, C., Boehm, B., Campo, E., Caricasole, A., et al. (2012). BLUEPRINT to decode the epigenetic signature written in blood. Nat. Biotechnol. *30*, 224–226.

Altorok, N., Coit, P., Hughes, T., Koelsch, K.A., Stone, D.U., Rasmussen, A., Radfar, L., Scofield, R.H., Sivils, K.L., Farris, A.D., and Sawalha, A.H. (2014).

Genome-wide DNA methylation patterns in naive CD4+ T cells from patients with primary Sjögren's syndrome. Arthritis Rheumatol. *66*, 731–739.

Arai, E., Chiku, S., Mori, T., Gotoh, M., Nakagawa, T., Fujimoto, H., and Kanai, Y. (2012). Single-CpG-resolution methylome analysis identifies clinicopathologically aggressive CpG island methylator phenotype clear cell renal cell carcinomas. Carcinogenesis *33*, 1487–1493.

Aryee, M.J., Jaffe, A.E., Corrada-Bravo, H., Ladd-Acosta, C., Feinberg, A.P., Hansen, K.D., and Irizarry, R.A. (2014). Minfi: a flexible and comprehensive Bioconductor package for the analysis of Infinium DNA methylation microarrays. Bioinformatics *30*, 1363–1369.

Barreau, O., Assié, G., Wilmot-Roussel, H., Ragazzon, B., Baudry, C., Perlemoine, K., René-Corail, F., Bertagna, X., Dousset, B., Hamzaoui, N., et al. (2013). Identification of a CpG island methylator phenotype in adrenocortical carcinomas. J. Clin. Endocrinol. Metab. *98*, E174–E184.

Benjamini, Y., and Hochberg, Y. (1995). Controlling the false discovery rate: A practical and powerful approach to multiple testing. J. R. Stat. Soc. Series B Stat. Methodol. *57*, 289–300.

Benjamini, Y., and Yekutieli, D. (2001). The control of the false discovery rate in multiple testing under dependency. Ann. Stat. *29*, 1165–1188.

Bibikova, M., Barnes, B., Tsan, C., Ho, V., Klotzle, B., Le, J.M., Delano, D., Zhang, L., Schroth, G.P., Gunderson, K.L., et al. (2011). High density DNA methylation array with single CpG site resolution. Genomics *98*, 288–295.

Birney, E., Smith, G.D., and Greally, J.M. (2016). Epigenome-wide Association Studies and the Interpretation of Disease -Omics. PLoS Genet. *12*, e1006105.

Coit, P., Jeffries, M., Altorok, N., Dozmorov, M.G., Koelsch, K.A., Wren, J.D., Merrill, J.T., McCune, W.J., and Sawalha, A.H. (2013). Genome-wide DNA methylation study suggests epigenetic accessibility and transcriptional poising of interferon-regulated genes in naïve CD4+ T cells from lupus patients. J. Autoimmun. *43*, 78–84.

Dorschner, M.O., Hawrylycz, M., Humbert, R., Wallace, J.C., Shafer, A., Kawamoto, J., Mack, J., Hall, R., Goldy, J., Sabo, P.J., et al. (2004). High-throughput localization of functional elements by quantitative chromatin profiling. Nat. Methods *1*, 219–225.

Dunham, I., Kulesha, E., Iotchkova, V., Morganella, S., and Birney, E. (2015). FORGE: A tool to discover cell specific enrichments of GWAS associated SNPs in regulatory regions. F1000Res.

Eckhardt, F., Lewin, J., Cortese, R., Rakyan, V.K., Attwood, J., Burger, M., Burton, J., Cox, T.V., Davies, R., Down, T.A., et al. (2006). DNA methylation profiling of human chromosomes 6, 20 and 22. Nat. Genet. *38*, 1378–1385.

ENCODE Project Consortium (2012). An integrated encyclopedia of DNA elements in the human genome. Nature *489*, 57–74.

Fang, F., Turcan, S., Rimner, A., Kaufman, A., Giri, D., Morris, L.G.T., Shen, R., Seshan, V., Mo, Q., Heguy, A., et al. (2011). Breast cancer methylomes establish an epigenomic foundation for metastasis. Sci. Transl. Med. *3*, 75ra25.

Farh, K.K.-H., Marson, A., Zhu, J., Kleinewietfeld, M., Housley, W.J., Beik, S., Shoresh, N., Whitton, H., Ryan, R.J.H., Shishkin, A.A., et al. (2015). Genetic and epigenetic fine mapping of causal autoimmune disease variants. Nature *518*, 337–343.

Hirohata, S., Yanagida, T., Itoh, K., Nakamura, H., Yoshino, S., Tomita, T., and Ochi, T. (1996). Accelerated generation of CD14+ monocyte-lineage cells from the bone marrow of rheumatoid arthritis patients. Arthritis Rheum. *39*, 836–843.

Houseman, E.A., Accomando, W.P., Koestler, D.C., Christensen, B.C., Marsit, C.J., Nelson, H.H., Wiencke, J.K., and Kelsey, K.T. (2012). DNA methylation arrays as surrogate measures of cell mixture distribution. BMC Bioinformatics *13*, 86.

Houseman, E.A., Kile, M.L., Christiani, D.C., Ince, T.A., Kelsey, K.T., and Marsit, C.J. (2016). Reference-free deconvolution of DNA methylation data and mediation by cell composition effects. BMC Bioinformatics *17*, 259.

Huynh, J.L., Garg, P., Thin, T.H., Yoo, S., Dutta, R., Trapp, B.D., Haroutunian, V., Zhu, J., Donovan, M.J., Sharp, A.J., and Casaccia, P. (2014). Epigenome-wide differences in pathology-free regions of multiple sclerosis-affected brains. Nat. Neurosci. *17*, 121–130.

Jaffe, A.E., and Irizarry, R.A. (2014). Accounting for cellular heterogeneity is critical in epigenome-wide association studies. Genome Biol. *15*, R31.

Kibriya, M.G., Raza, M., Jasmine, F., Roy, S., Paul-Brutus, R., Rahaman, R., Dodsworth, C., Rakibuz-Zaman, M., Kamal, M., and Ahsan, H. (2011). A genome-wide DNA methylation study in colorectal carcinoma. BMC Med. Genomics *4*, 50.

Köeferle, A., Stricker, S.H., and Beck, S. (2015). Brave new epigenomes: the dawn of epigenetic engineering. Genome Med. *7*, 59.

Kundaje, A., Meuleman, W., Ernst, J., Bilenky, M., Yen, A., Heravi-Moussavi, A., Kheradpour, P., Zhang, Z., Wang, J., Ziller, M.J., et al.; Roadmap Epigenomics Consortium (2015). Integrative analysis of 111 reference human epigenomes. Nature *518*, 317–330.

Laczmanska, I., Karpinski, P., Bebenek, M., Sedziak, T., Ramsey, D., Szmida, E., and Sasiadek, M.M. (2013). Protein tyrosine phosphatase receptor-like genes are frequently hypermethylated in sporadic colorectal cancer. J. Hum. Genet. *58*, 11–15.

Lavin, Y., Winter, D., Blecher-Gonen, R., David, E., Keren-Shaul, H., Merad, M., Jung, S., and Amit, I. (2014). Tissue-resident macrophage enhancer landscapes are shaped by the local microenvironment. Cell *159*, 1312–1326.

Li, H., Zheng, T., Chen, B., Hong, G., Zhang, W., Shi, T., Li, S., Ao, L., Wang, C., and Guo, Z. (2014). Similar blood-borne DNA methylation alterations in cancer and inflammatory diseases determined by subpopulation shifts in peripheral leukocytes. Br. J. Cancer *111*, 525–531.

Liang, L., and Cookson, W.O.C. (2014). Grasping nettles: cellular heterogeneity and other confounders in epigenome-wide association studies. Hum. Mol. Genet. *23* (*R1*), R83–R88.

Liu, Y., Aryee, M.J., Padyukov, L., Fallin, M.D., Hesselberg, E., Runarsson, A., Reinius, L., Acevedo, N., Taub, M., Ronninger, M., et al. (2013). Epigenome-wide association data implicate DNA methylation as an intermediary of genetic risk in rheumatoid arthritis. Nat. Biotechnol. *31*, 142–147.

Lowe, R., and Rakyan, V.K. (2013). Marmal-aid–a database for Infinium HumanMethylation450. BMC Bioinformatics *14*, 359.

Lowe, R., and Rakyan, V.K. (2014). Correcting for cell-type composition bias in epigenome-wide association studies. Genome Med. *6*, 23.

Lowe, R., Slodkowicz, G., Goldman, N., and Rakyan, V.K. (2015). The human blood DNA methylome displays a highly distinctive profile compared with other somatic tissues. Epigenetics *10*, 274–281.

Maurano, M.T., Humbert, R., Rynes, E., Thurman, R.E., Haugen, E., Wang, H., Reynolds, A.P., Sandstrom, R., Qu, H., Brody, J., et al. (2012). Systematic localization of common disease-associated variation in regulatory DNA. Science *337*, 1190–1195.

McGregor, K., Bernatsky, S., Colmegna, I., Hudson, M., Pastinen, T., Labbe, A., and Greenwood, C.M.T. (2016). An evaluation of methods correcting for cell-type heterogeneity in DNA methylation studies. Genome Biol. *17*, 84.

Michels, K.B., Binder, A.M., Dedeurwaerder, S., Epstein, C.B., Greally, J.M., Gut, I., Houseman, E.A., Izzi, B., Kelsey, K.T., Meissner, A., et al. (2013). Recommendations for the design and analysis of epigenome-wide association studies. Nat. Methods *10*, 949–955.

Paul, D.S., and Beck, S. (2014). Advances in epigenome-wide association studies for common diseases. Trends Mol. Med. *20*, 541–543.

Paul, D.S., Soranzo, N., and Beck, S. (2014). Functional interpretation of non-coding sequence variation: concepts and challenges. BioEssays *36*, 191–199.

Philibert, R., Hollenbeck, N., Andersen, E., Osborn, T., Gerrard, M., Gibbons, F.X., and Wang, K. (2015). A quantitative epigenetic approach for the assessment of cigarette consumption. Front. Psychol. *6*, 656.

Quinlan, A.R., and Hall, I.M. (2010). BEDTools: a flexible suite of utilities for comparing genomic features. Bioinformatics *26*, 841–842.

Rakyan, V.K., Down, T.A., Balding, D.J., and Beck, S. (2011). Epigenome-wide association studies for common human diseases. Nat. Rev. Genet. *12*, 529–541.

Schübeler, D. (2015). Function and information content of DNA methylation. Nature *517*, 321–326.

Singh, N., and Cohen, P.L. (2012). The T cell in Sjogren's syndrome: force majeure, not spectateur. J. Autoimmun. *39*, 229–233.

Slieker, R.C., Bos, S.D., Goeman, J.J., Bovée, J.V., Talens, R.P., van der Breggen, R., Suchiman, H.E.D., Lameijer, E.-W., Putter, H., van den Akker, E.B., et al. (2013). Identification and systematic annotation of tissue-specific differentially methylated regions using the Illumina 450k array. Epigenetics Chromatin *6*, 26.

Stadler, M.B., Murr, R., Burger, L., Ivanek, R., Lienert, F., Schöler, A., van Nimwegen, E., Wirbelauer, C., Oakeley, E.J., Gaidatzis, D., et al. (2011). DNA-binding factors shape the mouse methylome at distal regulatory regions. Nature *480*, 490–495.

Stergachis, A.B., Neph, S., Reynolds, A., Humbert, R., Miller, B., Paige, S.L., Vernot, B., Cheng, J.B., Thurman, R.E., Sandstrom, R., et al. (2013). Developmental fate and cellular maturity encoded in human regulatory DNA landscapes. Cell *154*, 888–903.

Teschendorff, A.E., Menon, U., Gentry-Maharaj, A., Ramus, S.J., Gayther, S.A., Apostolidou, S., Jones, A., Lechner, M., Beck, S., Jacobs, I.J., and Widschwendter, M. (2009). An epigenetic signature in peripheral blood predicts active ovarian cancer. PLoS ONE *4*, e8274.

Welter, D., MacArthur, J., Morales, J., Burdett, T., Hall, P., Junkins, H., Klemm, A., Flicek, P., Manolio, T., Hindorff, L., and Parkinson, H. (2014). The NHGRI GWAS Catalog, a curated resource of SNP-trait associations. Nucleic Acids Res. *42*, D1001–D1006.

Widschwendter, M., Fiegl, H., Egle, D., Mueller-Holzner, E., Spizzo, G., Marth, C., Weisenberger, D.J., Campan, M., Young, J., Jacobs, I., and Laird, P.W. (2007). Epigenetic stem cell signature in cancer. Nat. Genet. *39*, 157–158.

Wilhelm-Benartzi, C.S., Koestler, D.C., Karagas, M.R., Flanagan, J.M., Christensen, B.C., Kelsey, K.T., Marsit, C.J., Houseman, E.A., and Brown, R. (2013). Review of processing and analysis methods for DNA methylation array data. Br. J. Cancer *109*, 1394–1402.

Xavier, A.L., Menezes, J.R.L., Goldman, S.A., and Nedergaard, M. (2014). Fine-tuning the central nervous system: microglial modelling of cells and synapses. Philos. Trans. R. Soc. Lond. B Biol. Sci. *369*, 20130593.

Yin, Y., Choi, S.-C., Xu, Z., Perry, D.J., Seay, H., Croker, B.P., Sobel, E.S., Brusko, T.M., and Morel, L. (2015). Normalization of CD4+ T cell metabolism reverses lupus. Sci. Transl. Med. *7*, 274ra18.

Zou, J., Lippert, C., Heckerman, D., Aryee, M., and Listgarten, J. (2014). Epigenome-wide association studies without the need for cell-type composition. Nat. Methods *11*, 309–311.

Cell Reports, Volume *17*

Supplemental Information

eFORGE: A Tool for Identifying

Cell Type-Specific Signal in Epigenomic Data

Charles E. Breeze, Dirk S. Paul, Jenny van Dongen, Lee M. Butcher, John C. Ambrose, James E. Barrett, Robert Lowe, Vardhman K. Rakyan, Valentina Iotchkova, Mattia Frontini, Kate Downes, Willem H. Ouwehand, Jonathan Laperle, Pierre-Étienne Jacques, Guillaume Bourque, Anke K. Bergmann, Reiner Siebert, Edo Vellenga, Sadia Saeed, Filomena Matarese, Joost H.A. Martens, Hendrik G. Stunnenberg, Andrew E. Teschendorff, Javier Herrero, Ewan Birney, Ian Dunham, and Stephan Beck

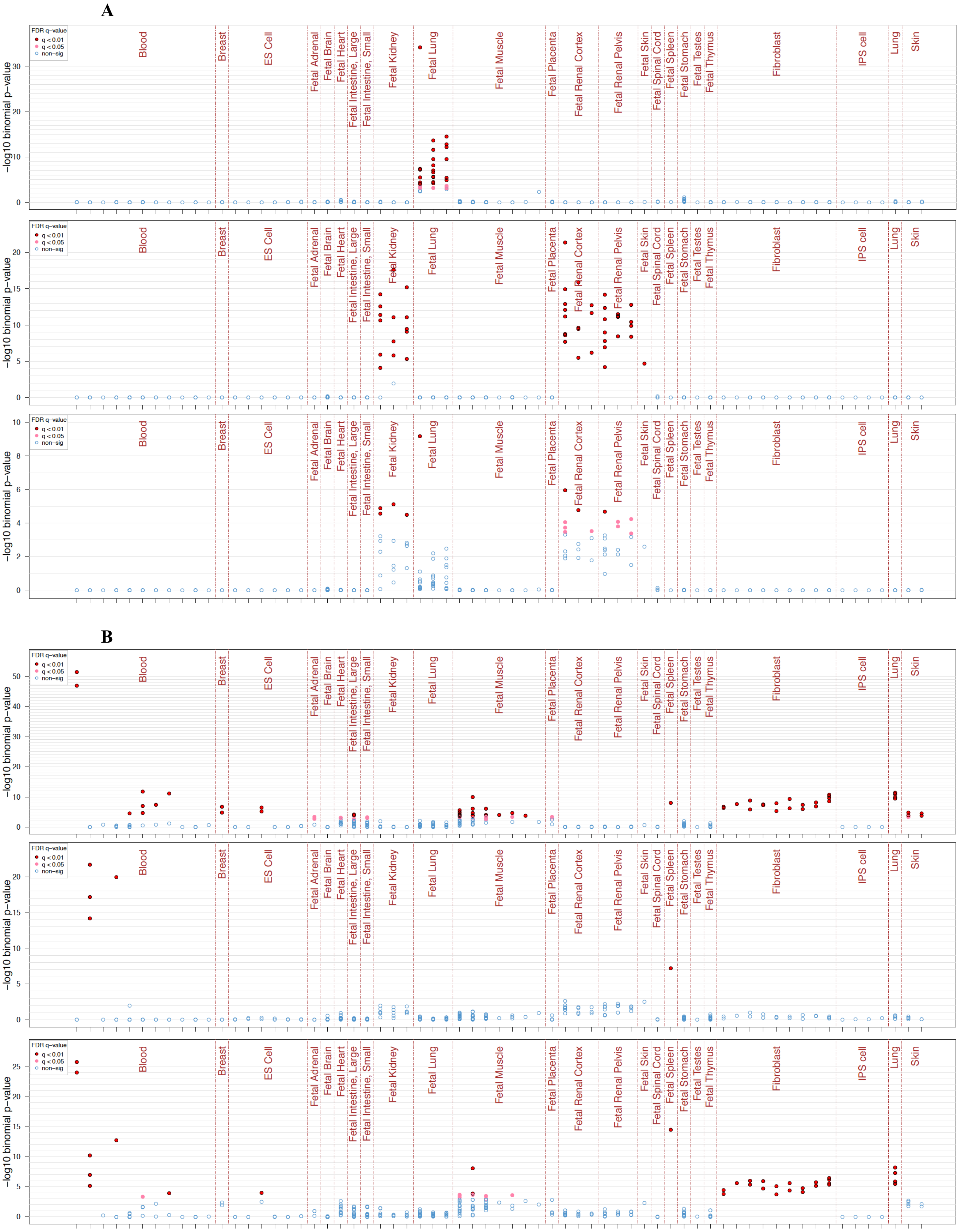
A
B
FDR q-value
q < 0.01
q < 0.05
non-sig
−log10 binomial p−value
Blood
Breast
ES Cell
Fetal Adrenal
Fetal Brain
Fetal Heart
Fetal Intestine, Large
Fetal Intestine, Small
Fetal Kidney
Fetal Lung
Fetal Muscle
Fetal Placenta
Fetal Renal Cortex
Fetal Renal Pelvis
Fetal Skin
Fetal Spinal Cord
Fetal Spleen
Fetal Stomach
Fetal Testes
Fetal Thymus
Fibroblast
IPS cell
Lung
Skin

Figure S1. eFORGE analysis of mixed DHS overlapping tDMP and cDMP sets. Related to Figure 2. [A]: eFORGE analysis of a mixed set of DHS-overlapping tDMPs. The top panel shows enrichment in lung for a list of 55 DHS-overlapping lung tDMPs. The middle panel shows enrichment in kidney for a set of 51 DHS-overlapping kidney tDMPs. The bottom panel shows mixed tissue-specific enrichment for lung and kidney for the merged list of 106 probes. **[B]:** eFORGE analysis of a mixed set of DHS-overlapping cDMPs. The top panel shows enrichment results from testing 148 monocyte specific DMPs, highlighting the CD14+ category. The middle panel shows enrichment results for 148 B cell specific DMPs, highlighting mainly the CD19+ and spleen categories. The bottom panel shows enrichment results for a mixed set of both cell type-specific probe sets. As evident from the mixed set, enrichments for both cell types are still present.

Intergenic region	Island	Shore_Shelf	NA
Within the 200 bp prior to a Transcription start site	Island	Shore_Shelf	NA
1st Exon	Island	Shore_Shelf	NA
Within the 1500 bp prior to a Transcription start site	Island	Shore_Shelf	NA
3' Untranslated region	Island	Shore_Shelf	NA
5' Untranslated region	Island	Shore_Shelf	NA
Gene Body	Island	Shore_Shelf	NA

Table S1. Data categories for background probe matching in eFORGE. Related to Figure 1. Column 1 indicates gene-centric category and columns 2, 3 and 4 indicate the three subsequent CpG Island-centric subcategories that separate probes from each gene-centric category.

IGR	Shore	21121
IGR	Island	22392
IGR	NA	57749
IGR	Shelf	18390
TSS200	Shore	9372
TSS200	Island	32996
TSS200	Shelf	857
TSS200	NA	9058
1stExon	Shore	2506
1stExon	Island	15581
1stExon	Shelf	368
1stExon	NA	4282
TSS1500	Shore	31022
TSS1500	Island	21610
TSS1500	Shelf	1685
TSS1500	NA	14667
3'UTR	Shore	3426
3'UTR	Island	1992
3'UTR	Shelf	1802
3'UTR	NA	10274
5'UTR	Shore	9460
5'UTR	Island	17581
5'UTR	NA	11855
5'UTR	Shelf	3789
Body	Shore	35160
Body	Island	38102
Body	Shelf	20253
Body	NA	68162
	Total	485512

Table S2. Numbers of probes in each background bin category in eFORGE. Related to Figure 1. The selection of each background probe matching each DMP in the test set is based on gene-centric and CpG island-centric annotation.

SetSize	N	p<0.05	p<0.01	q<0.05	q<0.01	F	p1f	p2f	q1f	q2f
5	625000	2418	190	0	0	5000	936	104	0	0
10	625000	23686	3602	23	0	5000	2829	943	11	0
15	625000	5721	597	0	0	5000	1338	283	0	0
20	625000	9313	1226	32	0	5000	1887	426	3	0
30	625000	13487	1591	4	0	5000	2274	542	1	0
40	625000	10877	1356	6	0	5000	1999	475	5	0
50	625000	13714	1554	16	0	5000	2222	570	4	0
100	625000	12973	1593	1	0	5000	2229	554	1	0

Table S3. Results from testing 5000 random probe sets for false positives on ENCODE data. Related to Figure 1. At q<0.01 we do not observe any false positives for any input test size (ranging between 5 and 100 probes).

SetSize	N	p<0.05	p<0.01	q<0.05	q<0.01	F	p1f	p2f	q1f	q2f
5	1495000	4161	313	0	0	5000	1013	142	0	0
10	1495000	54869	7930	264	0	5000	2998	1065	6	0
15	1495000	10519	904	0	0	5000	1544	316	0	0
20	1495000	16263	1570	1	0	5000	1894	431	1	0
30	1495000	28526	3279	0	0	5000	2214	602	0	0
40	1495000	22855	2557	129	0	5000	1997	496	2	0
50	1495000	30460	3430	1	0	5000	2341	672	1	0
100	1495000	23173	2480	1	0	5000	2051	510	1	0

Table S4. Results from testing 5000 random probe sets for false positives on Roadmap Epigenomics data. Related to Figure 1. At q<0.01 we do not observe any false positives for any input test size (ranging between 5 and 100 probes).

**Datatype**	**Assay**	**Celltype**	**Consortium**
**All (1785)**	0.49 {6}	0.08 {305}	-0.04 {3}
**H3K36me3 (255)**	1 {1}	0.32 {138}	-0.04 {2}
**H3K4me3 (292)**	1 {1}	0.25 {165}	-0.01 {2}
**H3K4me1 (239)**	1 {1}	0.37 {128}	0.06 {2}
**H3K27me3 (253)**	1 {1}	0.35 {134}	0.03 {2}
**H3K9me3 (238)**	1 {1}	0.26 {126}	0.07 {2}
**DNase-Seq (508)**	1 {1}	0.40 {183}	0.01 {3}

Table S5: Adjusted Rand Index (ARI) results for ENCODE, BLUEPRINT and Roadmap Epigenomics data clustering with GeEC. Related to Figure 1. An ARI score around zero means that the labels of a category are randomly distributed among clusters, while a positive value (max of 1) means that the labels of a category are enriched in a defined cluster. Numbers in ( ) represent the number of datasets in each group of datasets, while numbers in { } represent the number of different labels for each category and group. ARI results show that the contribution of "Consortium" is small, and the main labels for clustering are "Assay" and "Celltype".

Table S6: eFORGE BLUEPRINT sample data. Related to Figure 1. Sample data includes file name, lab of origin, type of data (all samples are DHS), cell name, tissue of origin, short cell name (for plotting), sample individual data (sex, age and disease status), sample URL and URL of original file. Samples include both primary cells and transformed cell lines. This table has been supplied as a Supplemental Excel file.

Study number	Number of samples	PMID	Disease/Trait	Platform	Analysed tissue	eFORGE tissue	eFORGE link
1	2442	23034122	Ageing in blood and brain **;***	27 and 450k	Whole blood, leukocytes, brain	Stem cell signal for 1000 probes from additional file 4	http://eforge.cs.ucl.ac.uk/eFORGE.v1.2/files/0xC8FF623812ED11E6B81D459BF8274B10/additional_file_4_ageing_EWAS.450k.erc2-DHS.chart.pdf
2	135	23093492	Adrenocortical carcinoma**	27k	Adrenocortical carcinoma tumours	Stem cell signal for probes from supplemental table 1 (362 probes) under 0.01	http://eforge.cs.ucl.ac.uk/eFORGE.v1.2/files/0x4D980C8425B311E6B2D23DDCF8274B10/index.html
3	910	23578854	Breast cancer	27k	blood	Mixed enrichment for several tissues for probes from Supplemental Table 1	http://eforge.cs.ucl.ac.uk/eFORGE.v1.2/files/0x439DAF16242011E6B310C6D3F8274B10/No_label_given.450k.erc2-DHS.chart.pdf
4	138	22610075	Clear cell renal cell carcinomas (RCCs)**	27k	29 normal renal cortex tissue, 109 tumor tissues and 107 matched adjacent normal tissue	Stem cell enrichment and other mixed enrichment for probes from supplemental table 2 (801 probes)	http://eforge.cs.ucl.ac.uk/eFORGE.v1.2/files/0x5550140A115211E6AF74B300F9274B10/No_label_given.450k.erc2-DHS.chart.pdf
5	367	21297937	Congenital Heart Defects (CHDs)	27k	blood	Mixed enrichment for probes from Table S1 (top 20 positive probes)	http://eforge.cs.ucl.ac.uk/eFORGE.v1.2/files/0xF3288FF2240A11E6A6E27FB6F8274B10/No_label_given.450k.erc2-DHS.chart.pdf
6	192	20687937	Diabetic nephropathy**	27k	Whole blood	Mixed enrichment for probes from supplemental table s1, with q values below 0.15	http://eforge.cs.ucl.ac.uk/eFORGE.v1.2/files/0x149CA7F4120E11E68EF480F5F8274B10/bell_diabetic_nefropathy_qvals_0_15.450k.erc2-DHS.chart.pdf
7	1230	22714737	Nonhematopoietic cancers and blood leukocyte profiles	27k	purified peripheral blood leukocyte subtypes from healthy donors	CD14 cell signal for probes from Supplemental Table 4	http://eforge.cs.ucl.ac.uk/eFORGE.v1.2/files/0x62FCC88C240D11E6A13229BAF8274B10/No_label_given.450k.erc2-DHS.chart.pdf
8	168	21453505	Parental age**	27k	cord blood	Blood signal for probes from table s5.	http://eforge.cs.ucl.ac.uk/eFORGE.v1.2/files/0x451A5388160D11

							E6945594A5F8274B10/No_label_given.450k.erc2-DHS.chart.pdf
9	201	21308978	Racial differences at birth**	27k	cord blood	Blood signal for "yes" probes from table s4:	http://eforge.cs.ucl.ac.uk/eFORGE.v1.2/files/0xBC47687C161411E6BF88B9AFF8274B10/No_label_given.450k.erc2-DHS.chart.pdf
10	255	23579546	Breast cancer	27k, 450k	breast	Blood and thymus signal for probes from Bre100 Supplemental Table 2A	http://eforge.cs.ucl.ac.uk/eFORGE.v1.2/files/0xC4946164242011E68EC468D4F8274B10/No_label_given.450k.erc2-DHS.chart.pdf
11	317	23526956	Clear cell renal cell carcinoma (ccRCC)	27k, 450k	kidney (Tumour and adjacent non-tumour tissue)	Kidney signal for top 200 methylated 450k probes from table S6	http://eforge.cs.ucl.ac.uk/eFORGE.v1.2/files/0xE35B9F46241F11E693220FD2F8274B10/No_label_given.450k.erc2-DHS.chart.pdf
12	656	23177740	Ageing***	450k	Whole blood	Stem cell signal for probes from table s5 model kidney, the largest TCGA category (183 Kidney samples, compared to 83 breast, 60 lung, and 42 skin samples):	http://eforge.cs.ucl.ac.uk/eFORGE.v1.2/files/0x2B74E1A8137B11E6AF31C3D2F8274B10/No_label_given.450k.erc2-DHS.chart.pdf
13	192	23332324	Child maltreatment***	450k	Salivary DNA	Mixed enrichment with blood skew for top 1000 probes by p-value from annex B, supplemental information	http://eforge.cs.ucl.ac.uk/eFORGE.v1.2/files/0x6B81FA2E12EA11E6A1B50A97F8274B10/appendix_B_child_abuse_EWAS.450k.erc2-DHS.chart.pdf
14	691	23334450	Rheumatoid arthritis	450k	blood	Blood enrichment, with other tissues, for probes from Supplemental Table 3	http://eforge.cs.ucl.ac.uk/eFORGE.v1.2/files/0xA38CF988241E11E68AF555D0F8274B10/No_label_given.450k.erc2-DHS.chart.pdf
15	184	23175441	Smoking status and cancer (breast and colon cancer)	450k	blood	Stomach enrichment for breast cancer comparison data	http://eforge.cs.ucl.ac.uk/eFORGE.v1.2/files/0x0E0FEA7E240E11E6A42C34BBF8274B10/No_label_given.450k.erc2-DHS.chart.pdf

16	1793	23691101	Tobacco smoking***	450k	Whole blood	Blood and thymus as main categories for probes from table s1	http://eforge.cs.ucl.ac.uk/eFORGE.v1.2/files/0x3C0CE156121211E68B61CBFAF8274B10/table_s1_tobacco.450k.erc2-DHS.chart.pdf
17	138	22232023	Tobacco smoking***	450k	119 lymphoblast DNA and 19 lung alveolarmacrophage DNA	Breast epithelial signature potentially signalling macrophage (BP confirmation) for probes from table 5	http://eforge.cs.ucl.ac.uk/eFORGE.v1.2/files/0x81C7A70412D911E687D76CFFF8274B10/No_label_given.450k.erc2-DHS.chart.pdf
18	153	23064414	Two subtypes of CLL; mutated or unmutated IGHV***	450k	Tumor samples and B cells from the controls	Mixed enrichment for top 1000 Met M-CLL / unmet U-CLL probes from supp table 5, with 3 out of 4 highest categories as blood subtypes, and also lung as enriched category	http://eforge.cs.ucl.ac.uk/eFORGE.v1.2/files/0xDA59362C0E2311E6A0BCC9DDF8274B10/index.html
19	261	20019873	Ovarian cancer**	27k	peripheral blood	CD14+ blood signal for a blood based EWAS for top 419 probes from supplemental table 2, agrees with article GSEA analysis results of immune specific genes, this is for 27k analysis	http://eforge.cs.ucl.ac.uk/eFORGE.v1.1/files/0x33752BF89CD311E4BEA3AC33AA596114
20	114	23666970	Thyroid cancer subtypes**	27k	114 snap-frozen thyroid samples	Cross-tissue mixed enrichment for first 1000 probes from supplemental table 2, highest category stem cell	http://eforge.cs.ucl.ac.uk/eFORGE.v1.2/files/0xA8797ECC0E0111E694B84EB2F8274B10/No_label_given.450k.erc2-DHS.chart.pdf

Table S7. List of analysed EWAS. Related to Figure 3 and Figure 4. Study information (columns 1-6), eFORGE enriched cell type (column 7) and eFORGE URL (column 8).

Supplemental files:

File S1. eFORGE datasets. Related to Figure 1.

File S2. tDMP datasets. Related to Figure 2.

File S3. Blood tDMPs. Related to Figure 2.

File S4. Kidney tDMPs. Related to Figure 2.

File S5. Lung tDMPs. Related to Figure 2.

Supplemental Experimental Procedures

DNase I Data

Hotspots from the ENCODE project (Thurman et al., 2012) were taken from ftp://ftp.ebi.ac.uk/pub/databases/ensembl/encode/integration_data_jan2011/byDataType/openchrom/jan2011/combined_hotspots/. Hotspots from the BLUEPRINT project were downloaded from https://blueprint.genomatix.de/grid/experiments/browse (**Table S6**). DNase I sequencing tag alignments from the Roadmap Epigenome Project were taken from http://www.genboree.org/EdaccData/Current-Release/experiment-sample/Chromatin_Accessibility/. The files that were used belong to the section of Gene Expression Omnibus (GEO) accession number GSE18927 available according to the data use embargo agreement. The Hotspot method (http://www.uwencode.org/proj/hotspot/) (John et al., 2011) (Sabo et al., 2004) was used to process the alignments, using the default parameters to give hotspot and peak files. Consolidated Roadmap Epigenomics DNase I hotspots and BroadPeak Histone mark data were retrieved from http://egg2.wustl.edu/roadmap/data/byFileType/peaks/consolidated/broadPeak/. Each of the datasets was assigned to the relevant cells and tissues using either consortium data or custom perl scripts from the decodings which are available from the ENCODE Data Coordination Centre tables (https://genome.ucsc.edu/encode/cellTypes.html) or from the BioSamples database sample group SAMEG31306 (http://www.ebi.ac.uk/biosamples/).

eFORGE probe weighting

Weights can be included in standalone eFORGE as follows. The initial weight can be set with the –weights flag and the step to generate subsequent weights can be set with the –step flag (see documentation at https://github.com/charlesbreeze/eFORGE/blob/eforge.v1.0/eforge.pl). Initial results show that weighting can improve certain enrichments, and better reflect the different significance (or effect size) of probes in the input list. However, we advise that the effect on the underlying statistics should be taken into consideration when introducing weights, and we advise caution when interpreting weighted results.

Supplemental References

John, S., Sabo, P.J., Thurman, R.E., Sung, M.-H., Biddie, S.C., Johnson, T.A., Hager, G.L., and Stamatoyannopoulos, J.A. (2011). Chromatin accessibility pre-determines glucocorticoid receptor binding patterns. Nat. Genet. *43*, 264–268.

Sabo, P.J., Hawrylycz, M., Wallace, J.C., Humbert, R., Yu, M., Shafer, A., Kawamoto, J., Hall, R., Mack, J., Dorschner, M.O., et al. (2004). Discovery of functional noncoding elements by digital analysis of chromatin structure. Proc. Natl. Acad. Sci. U. S. A. *101*, 16837–16842.

Thurman, R.E., Rynes, E., Humbert, R., Vierstra, J., Maurano, M.T., Haugen, E., Sheffield, N.C., Stergachis, A.B., Wang, H., Vernot, B., et al. (2012). The accessible chromatin landscape of the human genome. Nature *489*, 75–82.
